# IMA genome-F18: The re-identification of *Penicillium* genomes available in NCBI and draft genomes for *Penicillium* species from dry cured meat, *Penicilliumbiforme*, *P. brevicompactum*, *P. solitum*, and *P. cvjetkovicii*, *Pewenomyces kutranfy*, *Pew. lalenivora*, *Pew. tapulicola*, *Pew. kalosus*, *Teratosphaeria carnegiei*, and *Trichoderma atroviride* SC1

**DOI:** 10.1186/s43008-023-00121-w

**Published:** 2023-10-06

**Authors:** Cobus M. Visagie, Donato Magistà, Massimo Ferrara, Felipe Balocchi, Tuan A. Duong, Ales Eichmeier, David Gramaje, Janneke Aylward, Scott E. Baker, Irene Barnes, Sara Calhoun, Maria De Angelis, Jens C. Frisvad, Eliska Hakalova, Richard D. Hayes, Jos Houbraken, Igor V. Grigoriev, Kurt LaButti, Catarina Leal, Anna Lipzen, Vivian Ng, Jasmyn Pangilinan, Jakub Pecenka, Giancarlo Perrone, Anja Piso, Emily Savage, Milan Spetik, Michael J. Wingfield, Yu Zhang, Brenda D. Wingfield

**Affiliations:** 1 Department of Biochemistry, Genetics and Microbiology, Forestry and Agricultural Biotechnology Institute (FABI), University of Pretoria, Pretoria, South Africa; 2 Institute of Sciences of Food Production (ISPA), National Research Council (CNR), Via G. Amendola 122/O, 70126 Bari, Italy; 3 Mendeleum - Institute of Genetics, Mendel University in Brno, Valticka 334, 691 44 Lednice, Czech Republic; 4 Instituto de Ciencias de la Vid y del Vino (ICVV), Consejo Superior de Investigaciones Científicas - Universidad de la Rioja - Gobierno de La Rioja, Ctra. LO-20 Salida 13, Finca La Grajera, 26071 Logroño, Spain; 5 Department of Conservation Ecology and Entomology, Stellenbosch University, Matieland, Private Bag X1, Stellenbosch, 7602 South Africa; 6 Functional and Systems Biology Group, Environmental Molecular Sciences Division, Pacific Northwest National Laboratory, Richland, WA 99354 USA; 7 DOE Joint Bioenergy Institute, Emeryville, CA 94608 USA; 8 US Department of Energy Joint Genome Institute, Lawrence Berkeley National Laboratory, 1 Cyclotron Road, Berkeley, CA 94720 USA; 9 Department of Biotechnology and Biomedicine, Technical University of Denmark, Søltofts Plads, Building 221, 2800 Kgs Lyngby, Denmark; 10 Westerdijk Fungal Biodiversity Institute, Utrecht, The Netherlands; 11 Department of Plant and Microbial Biology, University of California Berkeley, 110 Koshland Hall, Berkeley, CA 94720 USA; 12 Department of Plant and Soil Sciences, FABI, University of Pretoria, Pretoria, South Africa; 13 Department of Soil, Plant and Food Sciences, University of Bari “Aldo Moro”, Via G. Amendola 165/a, 70126 Bari, Italy

## Introduction

Sequencing fungal genomes has now become very common and the list of genomes in this manuscript reflects this. Particularly relevant is that the first announcement is a re-identification of *Penicillium* genomes available on NCBI. The fact that more than 100 of these genomes have been deposited without the correct species names speak volumes to the fact that we must continue training fungal taxonomists and the importance of the International Mycological Association (after which this journal is named). When we started the genome series in 2013, one of the essential aspects was the need to have a phylogenetic tree as part of the manuscript. This came about as the result of a discussion with colleagues in NCBI who were trying to deal with the very many incorrectly identified bacterial genomes (at the time) which had been submitted to NCBI. We are now in the same position with fungal genomes. Sequencing a fungal genome is all too easy but providing a correct species name and ensuring that the fungus has in fact been correctly identified seems to be more difficult. We know that there are thousands of fungi which have not yet been described. The availability of sequence data has made identification of fungi easier but also serves to highlight the need to have a fungal taxonomist in the project to make sure that mistakes are not made.

## IMA GENOME‐F 18A

### The re-identification of *Penicillium* genomes available in NCBI

#### Introduction

*Penicillium* and its 536 accepted species represent one of the most commonly occurring and important fungal genera (Houbraken et al. 2020; Visagie et al. 2014). In recent years, whole genome sequencing efforts have increased and hundreds of *Penicillium* genomes are publicly available in the NCBI genome database (https://www.ncbi.nlm.nih.gov/datasets/genome). The study of these genomes is important, for example, to gain a better understanding of the biology of certain species. However, these studies and their communication depend on the use of the correct name of the genomes and the conclusions drawn from them. Analyses such as genome comparisons based on incorrect identifications lead to incorrect conclusions. The problem of misidentified genomes has already been highlighted by Houbraken et al. (2021), who also made several recommendations to prevent misidentifications in future. To support future studies using the genomes currently available in NCBI with the name *Penicillium*, we re-identify the genomes here using the modern taxonomy of the genus as published in Houbraken et al. (2020) who published an accepted species list and an updated subgeneric classification at the subgenus, section and series levels.

#### Materials and methods

A *Penicillium* reference dataset was compiled mainly based on the most recent taxonomy and accepted species list published by Houbraken et al. (2020). The six gene regions included in the dataset were beta-tubulin (*BenA*), calmodulin (*CaM*), RNA polymerase II second largest subunit (*RPB2*), RNA polymerase II largest subunit *(RPB1*), the subunit of the cytosolic chaperonin Cct ring complex (*Cct8*), and *Tsr1*, the protein required for processing 20S pre-rRNA in the cytoplasm. These gene regions were extracted from genomes downloaded for *Penicillium* from the NCBI Genome Portal using Geneious Prime v. 2023.1.2 and included in the dataset.

In our multi-gene phylogenetic analysis, each gene region was treated as separate partitions and introns and exons were taken into consideration where appropriate. Datasets were aligned using MAFFT v. 7.490 with the G-INS-i option (Katoh and Standley 2013). Alignments were trimmed or adjusted as needed and then concatenated in Geneious Prime. The General Time Reversible nucleotide substitution model with gamma distribution with invariant site (GTR + G + I) was chosen for all partitions. Maximum likelihood trees were calculated in IQ-tree v. 2.1.3 (Minh et al. 2020), subsequently visualised in TreeViewer v. 2.0.1 (https://treeviewer.org/) and edited in Affinity Publisher v. 2 (Serif (Europe), Nottingham, UK). The reference datasets, alignments and tree files were uploaded to the University of Pretoria research data repository hosted on Figshare (https://www.doi.org/10.25403/UPresearchdata.24004071).

#### Results and discussion

**Fig. 1:**
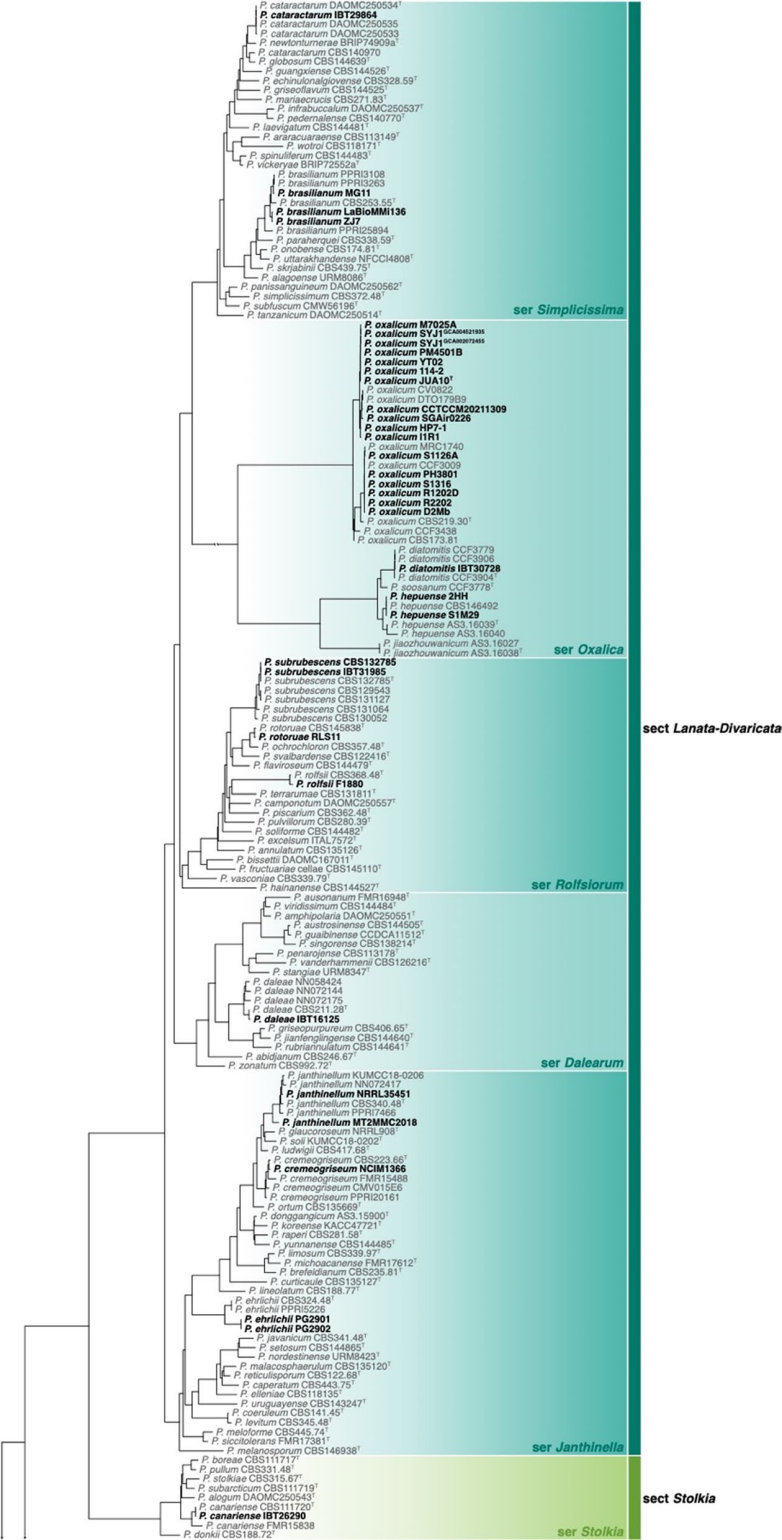

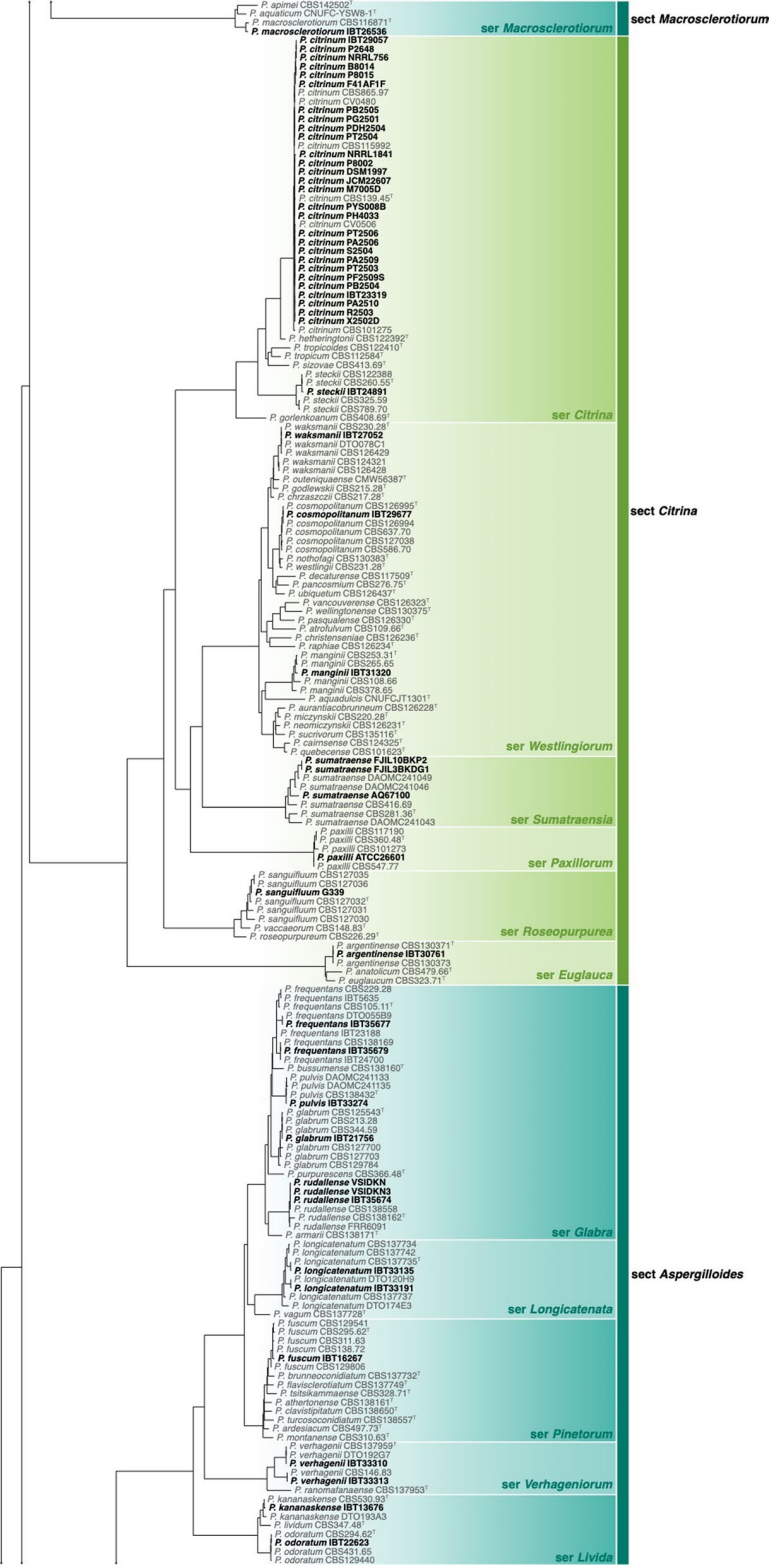

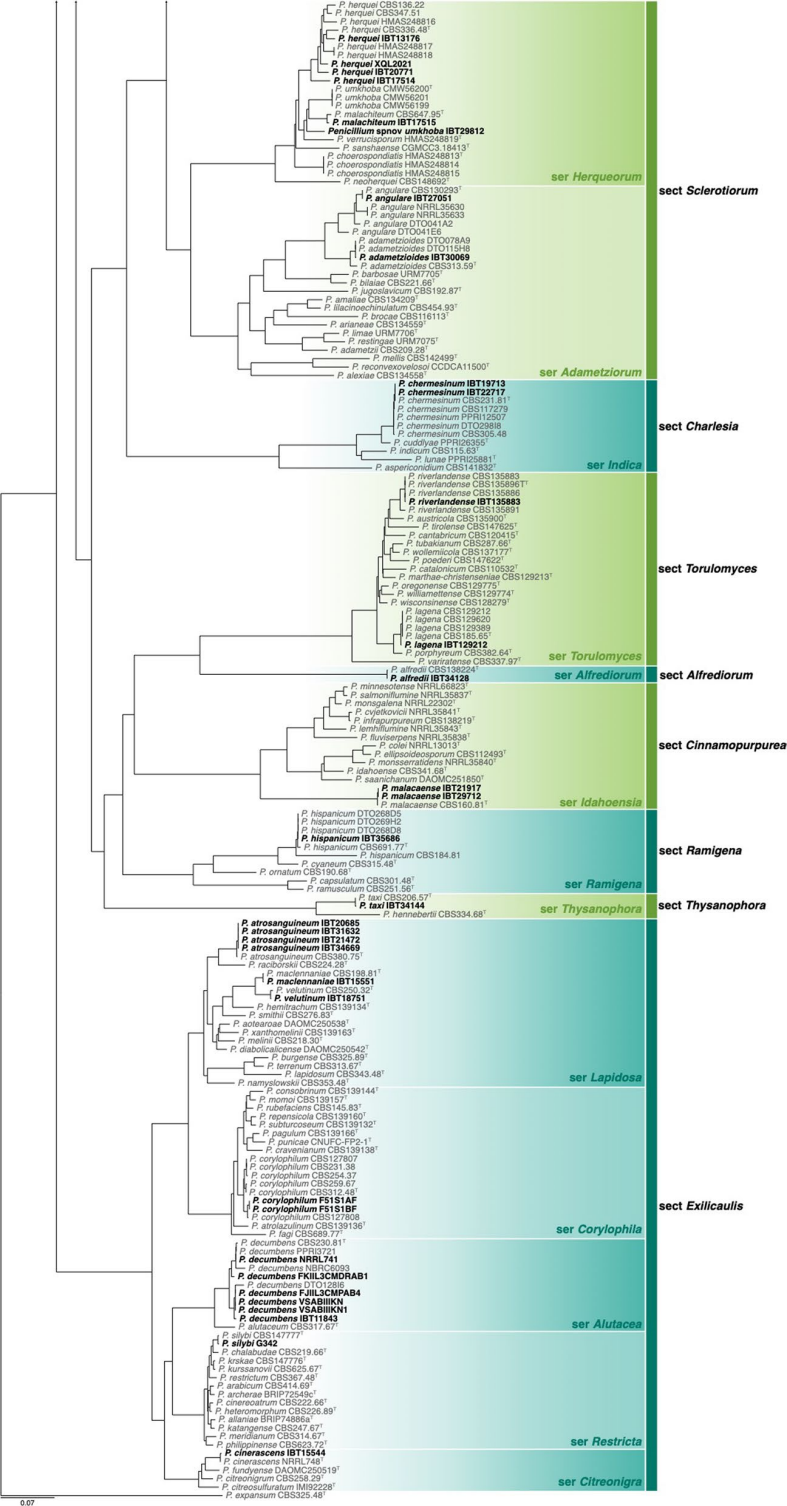
Phylogenetic tree of *Penicillium* subgenus *Aspergilloides* based on a concatenated dataset of *BenA*, *CaM*, *Cct8*, *RPB1*, *RPB2* and *Tsr1*. Data obtained from available NCBI genomes appear in bold black text. Reference sequences appear in grey text. Ex-type strains are indicated by superscript T. The tree was rooted to *P. expansum*.

**Fig. 2:**
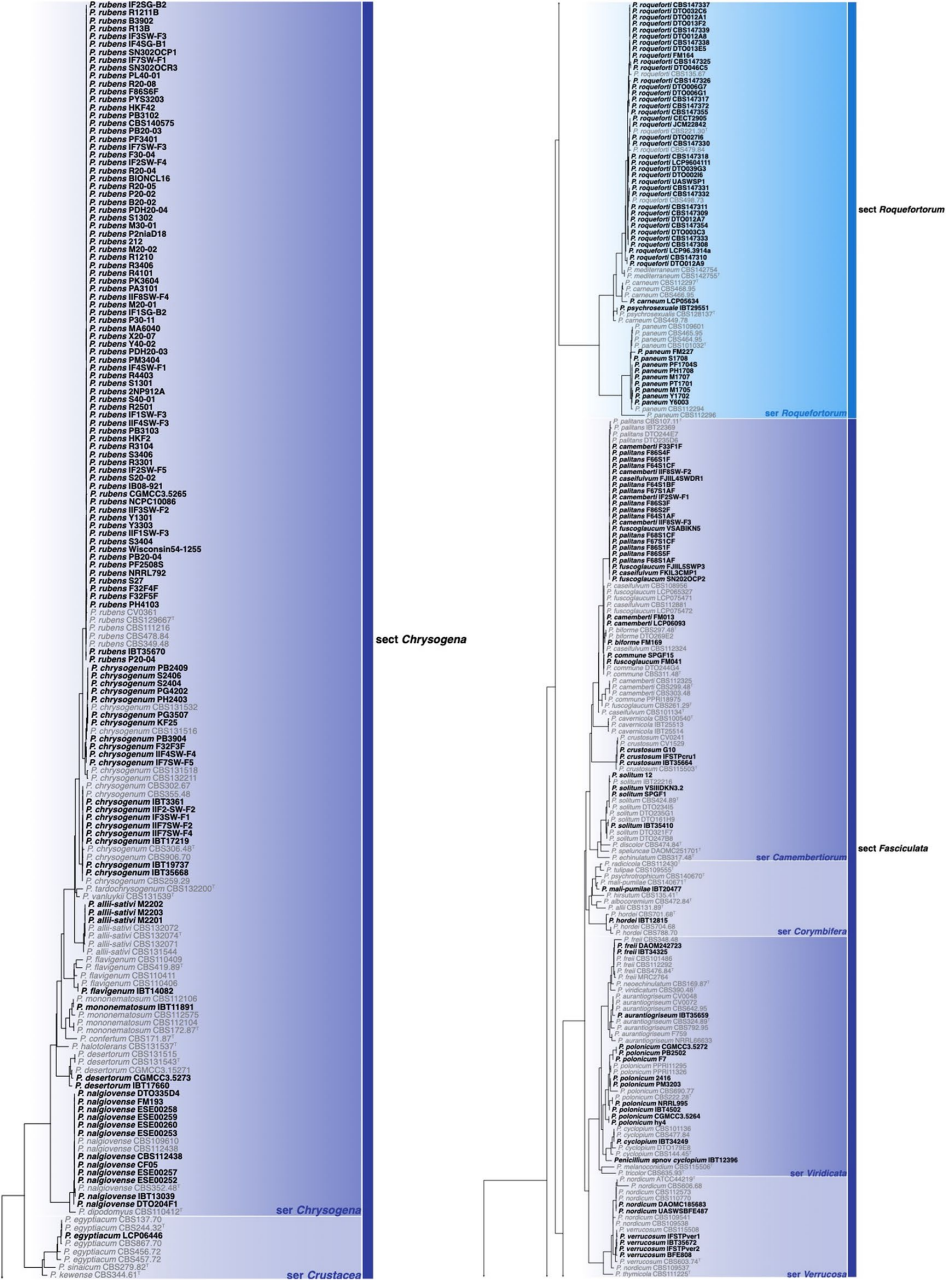

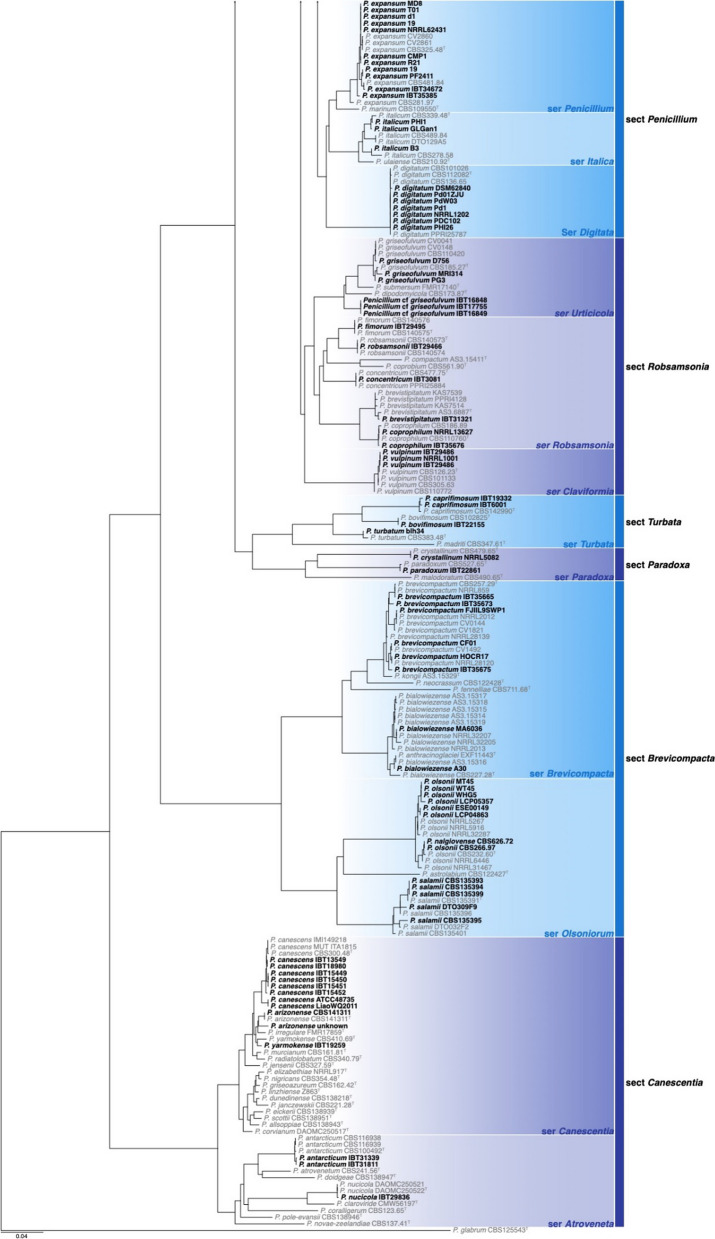
Phylogenetic tree of *Penicillium* subgenus *Penicillium* based on a concatenated dataset of *BenA*, *CaM*, *Cct8*, *RPB1*, *RPB2* and *Tsr1*. Data obtained from available NCBI genomes appear in bold black text. Reference sequences appear in grey text. Ex-type strains are indicated by superscript T. The tree was rooted to *P. glabrum*.

Of the 426 genomes analysed in this study, 281 were correctly identified, 87 were misnamed, 12 were misidentified and 33 were submitted as *Penicillium* without a species name (see Table 1, Additional file 1: Table 1 and Figs 1, 2). Of the correctly identified strains, 27 resolved in the *P. camemberti* species complex in the series *Camembertiorum*. This group is economically important and is typically used for the production of cheese like brie or camembert (Thom 1906). Taxonomically, this group and its six accepted species needs to be revised, but is complicated due to several past domestications (Ropars et al. 2020a, b). As there is little to no phylogenetic variation to guide identifications, we accept the name under which genomes from this group were submitted. Of the misidentified genomes, five belong to different genera including: GCA_023625675, which we believe to be a *Candida* species; GCA_023627405, which belongs to *Aspergillus ustus*; GCA_011750695, which belongs to *Talaromyces minnesotensis*; and GCA_002382835 and GCA_002382855, which belong to *Talaromyces pinophilus*. Six genomes were labelled with old names that have been synonymised, including: GCA_028828285 belonging to *P. solitum* (= *P. majusculim*) (Frisvad and Samson 2004)); GCA_025586815 belonging to *P. desertorum* (= *P. glycyrrhizacola*); GCA_015585885, GCA_015586035 and GCA_015585865 belonging to *P. chrysogenum* (= *P. griseoroseum*) (Houbraken et al. 2012); and GCA_028829675 belonging to *P. glabrum* (= *P. tannophilum*) (Houbraken et al. 2014). GCA_028974045 was submitted as a potential new species closely related to *P. viridicatum* and is identical to the recently described *P. mali-pumilae* (Hyde et al. 2019)*.* Based on our analyses, we have identified three new species, including: GCA_028828675 in section *Sclerotiora* series *Herqueorum*; GCA_028827225 in section *Fasciculata* series *Viridicata*; and GCA_028826995, GCA_028974015 and GCA_028827235 in section *Robsamsonia* series *Urticicola*. Among the misidentified genomes were 12 that belong to different sections, including: GCA_000943775 and GCA_000943765 belonging to *P. canescens* in section *Canescentia* (not *P. capsulatum* in section *Ramigena*); GCA_015585765 and GCA_015585785 belonging to *P. chrysogenum* in section *Chrysogena* (not *P. dipodomyicola* in section *Robsamsonia*); GCA_028828875 and GCA_028826875 belonging to *P. malacaense* in section *Idahoensia* (not *P. capsulatum* in section *Ramigena*); GCA_015585975 belonging to *P. rubens* in section *Chrysogena* (not *P. dipodomyicola* in section *Robsamsonia*); GCA_020284065, GCA_019827435 and GCA_019828795 belonging to *P. rubens* in section *Chrysogena* (not *P. fimorum* in section *Robsamsonia*); GCA_015585905 belonging to *P. rubens* in section *Chrysogena* (not *P. polonicum* in section *Fasciculata*); and GCA_019804565 belonging to *P. solitum* in section *Fasciculata* (not *P. robsamsonii* in section *Robsamsonia*). We consider 87 genomes misnamed, with the submitted name being classified in the same series as our re-identified name. An example of this is the large number of genomes submitted as *P. chrysogenum* that belong to its closest relative, *P. rubens* in the series *Chrysogena*.

**Table 1:** Summary of genomes re-identified during this study. See Additonal file 1: Table 1 for the full list of strains analysed during this study.

Assembly acc	WGS project acc	Current species name	Subgenus	Section	Series	Strain	Submitted name	Note
GCA_023627405	JAMAHF01	*Aspergillus ustus*	*Nidulantes*	*Usti*	*Usti*	R2504	*Penicillium* species	Misidentified (Incorrect genus)
GCA_023625675	JAMAFL01	*Candida* species	*–*	*–*	*–*	R2202	*Penicillium* species	Misidentified (Incorrect genus)
GCA_028826755	JAPQKH01	*P. adametzioides*	*Aspergilloides*	*Sclerotiorum*	*Adametziorum*	IBT30069	*P. angulare*	Misidentified
GCA_023624315	JAMACP01	*P. allii-sativi*	*Penicillium*	*Chrysogena*	*Chrysogena*	M2201	*P. chrysogenum*	Misidentified
GCA_023627315	JAMAFH01	*P. allii-sativi*	*Penicillium*	*Chrysogena*	*Chrysogena*	M2202	*P. chrysogenum*	Misidentified
GCA_025768175	JAMFOR01	*P. allii-sativi*	*Penicillium*	*Chrysogena*	*Chrysogena*	M2203	*P. chrysogenum*	Misidentified
GCA_003138045	QAGG01	*P. bialowiezense*	*Penicillium*	*Brevicompacta*	*Brevicompacta*	MA6036	*Penicillium* species	Identified to genus
GCA_005250745	RFFF02	*P. brevicompactum*	*Penicillium*	*Brevicompacta*	*Brevicompacta*	CF01	*Penicillium* species	Identified to genus
GCA_002072405	MDDG01	*P. brevistipitatum*	*Penicillium*	*Robsamsonia*	*Robsamsonia*	IBT31321	*P. coprophilum*	Misidentified
GCA_000943765	JPLQ01	*P. canescens*	*Penicillium*	*Canescentia*	*Canescentia*	ATCC48735	*P. capsulatum*	Misidentified (wrong section)
GCA_000943775	JPLR01	*P. canescens*	*Penicillium*	*Canescentia*	*Canescentia*	LiaoWQ2011	*P. capsulatum*	Misidentified (wrong section)
GCA_028827535	JAQJAA01	*P. caprifimosum*	*Penicillium*	*Turbata*	*Turbata*	IBT19332	*Penicillium* species	Identified to genus
GCA_028827425	JAQJAB01	*P. caprifimosum*	*Penicillium*	*Turbata*	*Turbata*	IBT6001	*Penicillium* species	Identified to genus
GCA_015586035	JACSPC01	*P. chrysogenum*	*Penicillium*	*Chrysogena*	*Chrysogena*	IF3SW-F1	*P. griseoroseum*	Synonym
GCA_015585885	JACSOW01	*P. chrysogenum*	*Penicillium*	*Chrysogena*	*Chrysogena*	IF7SW-F5	*P. griseoroseum*	Synonym
GCA_015585865	JACSOT01	*P. chrysogenum*	*Penicillium*	*Chrysogena*	*Chrysogena*	IIF4SW-F4	*P. griseoroseum*	Synonym
GCA_015585785	JACSOR01	*P. chrysogenum*	*Penicillium*	*Chrysogena*	*Chrysogena*	IIF7SW-F2	*P. dipodomyicola*	Misidentified (wrong section)
GCA_015585765	JACSOQ01	*P. chrysogenum*	*Penicillium*	*Chrysogena*	*Chrysogena*	IIF7SW-F4	*P. dipodomyicola*	Misidentified (wrong section)
GCA_028827645	JAQJAC01	*P. citrinum*	*Aspergilloides*	*Citrina*	*Citrina*	IBT29057	*P. hetheringtonii*	Misidentified
GCA_018340795	JADDUG01	*P. citrinum*	*Aspergilloides*	*Citrina*	*Citrina*	P2648	*P. steckii*	Misidentified
GCA_003800485	PUHX02	*P. commune*	*Penicillium*	*Fasciculata*	*Camembertiorum*	SPGF15	*Penicillium* species	Identified to genus
GCA_002369805	NPFE01	*P. cremeogriseum*	*Aspergilloides*	*Lanata-Divaricata*	*Janthinella*	NCIM1366	*P. janthinellum*	Misidentified
GCA_028828185	JAQKAP01	*P. cyclopium*	*Penicillium*	*Fasciculata*	*Viridicata*	IBT34249	*P. viridicatum*	Misidentified
GCA_019775275	JACWGB01	*P. decumbens*	*Aspergilloides*	*Exilicaulis*	*Alutacea*	VSABIIIKN	*Penicillium* species	Identified to genus
GCA_019775305	JACWGC01	*P. decumbens*	*Aspergilloides*	*Exilicaulis*	*Alutacea*	VSABIIIKN1	*Penicillium* species	Identified to genus
GCA_025586815	JANFQT01	*P. desertorum*	*Penicillium*	*Chrysogena*	*Chrysogena*	CGMCC3.5273	*P. glycyrrhizacola*	Synonym
GCA_023626475	JAMADV01	*P. ehrlichii*	*Aspergilloides*	*Lanata-Divaricata*	*Janthinella*	PG2901	*P. janthinellum*	Misidentified
GCA_023626455	JAMADU01	*P. ehrlichii*	*Aspergilloides*	*Lanata-Divaricata*	*Janthinella*	PG2902	*P. janthinellum*	Misidentified
GCA_028828275	JAQIZY01	*P. frequentans*	*Aspergilloides*	*Aspergilloides*	*Glabra*	IBT35677	*P. glabrum*	Misidentified
GCA_028827865	JAQIZZ01	*P. frequentans*	*Aspergilloides*	*Aspergilloides*	*Glabra*	IBT35679	*P. glabrum*	Misidentified
GCA_028828865	JAQJZQ01	*P. fuscum*	*Aspergilloides*	*Aspergilloides*	*Pinetorum*	IBT16267	*Penicillium* species	Identified to genus
GCA_028829675	JAQKAK01	*P. glabrum*	*Aspergilloides*	*Aspergilloides*	*Glabra*	IBT21756	*P. tannophilum*	Synonym
GCA_014839855	WIWU01	*P. hepuense*	*Aspergilloides*	*Lanata-Divaricata*	*Oxalica*	2HH	*P. ucsense*	Misidentified
GCA_014839625	WIWV01	*P. hepuense*	*Aspergilloides*	*Lanata-Divaricata*	*Oxalica*	S1M29	*P. ucsense*	Misidentified
GCA_028827775	JAQJAM01	*P. herquei*	*Aspergilloides*	*Sclerotiorum*	*Herqueorum*	IBT13176	*P. malachiteum*	Misidentified
GCA_028828335	JAQJAN01	*P. herquei*	*Aspergilloides*	*Sclerotiorum*	*Herqueorum*	IBT17514	*P. malachiteum*	Misidentified
GCA_003852855	PZKB01	*P. janthinellum*	*Aspergilloides*	*Lanata-Divaricata*	*Janthinella*	MT2MMC2018	*Penicillium* species	Identified to genus
GCA_028828245	JAQJAG01	*P. kananaskense*	*Aspergilloides*	*Aspergilloides*	*Livida*	IBT13676	*P. lividum*	Misidentified
GCA_028826875	JAPQKO01	*P. malacaense*	*Aspergilloides*	*Cinnamopurpurea*	*Idahoensia*	IBT21917	*P. capsulatum*	Misidentified (wrong section)
GCA_028828875	JAQJZR01	*P. malacaense*	*Aspergilloides*	*Cinnamopurpurea*	*Idahoensia*	IBT29712	*P. capsulatum*	Misidentified (wrong section)
GCA_028974045	JAPQKQ01	*P. mali-pumilae*	*Penicillium*	*Fasciculata*	*Corymbifera*	IBT20477	*Penicillium* cf* viridicatum*	Misidentified
GCA_002916455	PKQL01	*P. nalgiovense*	*Penicillium*	*Chrysogena*	*Chrysogena*	CF05	*Penicillium* species	Identified to genus
GCA_029142755	JARFLT01	*P. olsonii*	*Penicillium*	*Brevicompacta*	*Olsoniorum*	MT45	*Penicillium* species	Identified to genus
GCA_029142805	JARFLS01	*P. olsonii*	*Penicillium*	*Brevicompacta*	*Olsoniorum*	WT45	*Penicillium* species	Identified to genus
GCA_023626495	JAMAHB01	*P. oxalicum*	*Aspergilloides*	*Lanata-Divaricata*	*Oxalica*	D2Mb	*Penicillium* species	Identified to genus
GCA_023626535	JAMAHD01	*P. oxalicum*	*Aspergilloides*	*Lanata-Divaricata*	*Oxalica*	PH3801	*Penicillium* species	Identified to genus
GCA_023626555	JAMAHA01	*P. oxalicum*	*Aspergilloides*	*Lanata-Divaricata*	*Oxalica*	R1202D	*Penicillium* species	Identified to genus
GCA_025768115	JAMFOT01	*P. oxalicum*	*Aspergilloides*	*Lanata-Divaricata*	*Oxalica*	R2202	*Penicillium* species	Identified to genus
GCA_023626515	JAMAHC01	*P. oxalicum*	*Aspergilloides*	*Lanata-Divaricata*	*Oxalica*	S1126A	*Penicillium* species	Identified to genus
GCA_023626615	JAMAHE01	*P. oxalicum*	*Aspergilloides*	*Lanata-Divaricata*	*Oxalica*	S1316	*Penicillium* species	Identified to genus
GCA_022985105	JAAVMA01	*P. rotoruae*	*Aspergilloides*	*Lanata-Divaricata*	*Rolfsiorum*	RLS11	*P. ochrochloron*	Misidentified
GCA_023626975	JAMAEP01	*P. rubens*	*Penicillium*	*Chrysogena*	*Chrysogena*	2NP912A	*P. chrysogenum*	Misidentified
GCA_023626635	JAMAEB01	*P. rubens*	*Penicillium*	*Chrysogena*	*Chrysogena*	B20-02	*P. chrysogenum*	Misidentified
GCA_023626855	JAMAEG01	*P. rubens*	*Penicillium*	*Chrysogena*	*Chrysogena*	B3902	*P. chrysogenum*	Misidentified
GCA_028891605	JAKRWH01	*P. rubens*	*Penicillium*	*Chrysogena*	*Chrysogena*	BIONCL16	*P. chrysogenum*	Misidentified
GCA_020284065	JAILXA01	*P. rubens*	*Penicillium*	*Chrysogena*	*Chrysogena*	CBS140575	*P. fimorum*	Misidentified (wrong section)
GCA_025590035	JANFQV01	*P. rubens*	*Penicillium*	*Chrysogena*	*Chrysogena*	CGMCC3.5265	*P. chrysogenum*	Misidentified
GCA_023627295	JAMAFF01	*P. rubens*	*Penicillium*	*Chrysogena*	*Chrysogena*	F30-04	*P. chrysogenum*	Misidentified
GCA_015586305	JADBGY01	*P. rubens*	*Penicillium*	*Chrysogena*	*Chrysogena*	F32F4F	*P. chrysogenum*	Misidentified
GCA_015586295	JADBGZ01	*P. rubens*	*Penicillium*	*Chrysogena*	*Chrysogena*	F32F5F	*P. chrysogenum*	Misidentified
GCA_002000375	MUXA01	*P. rubens*	*Penicillium*	*Chrysogena*	*Chrysogena*	HKF2	*Penicillium* species	Identified to genus
GCA_002080375	MWKT01	*P. rubens*	*Penicillium*	*Chrysogena*	*Chrysogena*	HKF42	*P. chrysogenum*	Misidentified
GCA_000801355	JPDR01	*P. rubens*	*Penicillium*	*Chrysogena*	*Chrysogena*	IB08-921	*P. chrysogenum*	Misidentified
GCA_015586425	JADBGS01	*P. rubens*	*Penicillium*	*Chrysogena*	*Chrysogena*	IF1SG-B2	*P. chrysogenum*	Misidentified
GCA_015586135	JACSPG01	*P. rubens*	*Penicillium*	*Chrysogena*	*Chrysogena*	IF1SW-F3	*P. chrysogenum*	Misidentified
GCA_015586415	JADBGT01	*P. rubens*	*Penicillium*	*Chrysogena*	*Chrysogena*	IF2SG-B2	*P. chrysogenum*	Misidentified
GCA_015586075	JACSPE01	*P. rubens*	*Penicillium*	*Chrysogena*	*Chrysogena*	IF2SW-F4	*P. chrysogenum*	Misidentified
GCA_015586055	JACSPD01	*P. rubens*	*Penicillium*	*Chrysogena*	*Chrysogena*	IF2SW-F5	*P. chrysogenum*	Misidentified
GCA_015586015	JACSPB01	*P. rubens*	*Penicillium*	*Chrysogena*	*Chrysogena*	IF3SW-F3	*P. chrysogenum*	Misidentified
GCA_015586395	JADBGU01	*P. rubens*	*Penicillium*	*Chrysogena*	*Chrysogena*	IF4SG-B1	*P. chrysogenum*	Misidentified
GCA_015585965	JACSPA01	*P. rubens*	*Penicillium*	*Chrysogena*	*Chrysogena*	IF4SW-F1	*P. chrysogenum*	Misidentified
GCA_015585955	JACSOZ01	*P. rubens*	*Penicillium*	*Chrysogena*	*Chrysogena*	IF7SW-F1	*P. chrysogenum*	Misidentified
GCA_015585975	JACSOY01	*P. rubens*	*Penicillium*	*C hrysogena*	*Chrysogena*	IF7SW-F3	*P. dipodomyicola*	Misidentified (wrong section)
GCA_015585905	JACSOV01	*P. rubens*	*Penicillium*	*Chrysogena*	*Chrysogena*	IIF1SW-F3	*P. polonicum*	Misidentified (wrong section)
GCA_015585875	JACSOU01	*P. rubens*	*Penicillium*	*Chrysogena*	*Chrysogena*	IIF3SW-F2	*P. chrysogenum*	Misidentified
GCA_015585855	JACTVK01	*P. rubens*	*Penicillium*	*Chrysogena*	*Chrysogena*	IIF4SW-F3	*P. chrysogenum*	Misidentified
GCA_015585735	JACSON01	*P. rubens*	*Penicillium*	*Chrysogena*	*Chrysogena*	IIF8SW-F4	*P. chrysogenum*	Misidentified
GCA_023626815	JAMAEH01	*P. rubens*	*Penicillium*	*Chrysogena*	*Chrysogena*	M20-01	*P. chrysogenum*	Misidentified
GCA_023627355	JAMAFJ01	*P. rubens*	*Penicillium*	*Chrysogena*	*Chrysogena*	M20-02	*P. chrysogenum*	Misidentified
GCA_023627035	JAMAET01	*P. rubens*	*Penicillium*	*Chrysogena*	*Chrysogena*	M30-01	*P. chrysogenum*	Misidentified
GCA_003138025	QAGI01	*P. rubens*	*Penicillium*	*Chrysogena*	*Chrysogena*	MA6040	*Penicillium* species	Identified to genus
GCA_000523475	APKG01	*P. rubens*	*Penicillium*	*Chrysogena*	*Chrysogena*	NCPC10086	*P. chrysogenum*	Misidentified
GCA_027569345	JAPDEX01	*P. rubens*	*Penicillium*	*Chrysogena*	*Chrysogena*	NRRL792	*P. chrysogenum*	Misidentified
GCA_023624235	JAMACS01	*P. rubens*	*Penicillium*	*Chrysogena*	*Chrysogena*	P20-02	*P. chrysogenum*	Misidentified
GCA_023624435	JAMACH01	*P. rubens*	*Penicillium*	*Chrysogena*	*Chrysogena*	P20-04	*P. chrysogenum*	Misidentified
GCA_000710275	JMSF01	*P. rubens*	*Penicillium*	*Chrysogena*	*Chrysogena*	P2niaD18	*P. chrysogenum*	Misidentified
GCA_023624455	JAMACI01	*P. rubens*	*Penicillium*	*Chrysogena*	*Chrysogena*	P30-11	*P. chrysogenum*	Misidentified
GCA_023626755	JAMAEF01	*P. rubens*	*Penicillium*	*Chrysogena*	*Chrysogena*	PA3101	*P. chrysogenum*	Misidentified
GCA_023626715	JAMAEC01	*P. rubens*	*Penicillium*	*Chrysogena*	*Chrysogena*	PB20-03	*P. chrysogenum*	Misidentified
GCA_025768475	JAMFOQ01	*P. rubens*	*Penicillium*	*Chrysogena*	*Chrysogena*	PB20-04	*P. chrysogenum*	Misidentified
GCA_023624375	JAMACL01	*P. rubens*	*Penicillium*	*Chrysogena*	*Chrysogena*	PB3102	*P. chrysogenum*	Misidentified
GCA_023626675	JAMAEA01	*P. rubens*	*Penicillium*	*Chrysogena*	*Chrysogena*	PB3103	*P. chrysogenum*	Misidentified
GCA_023627015	JAMAES01	*P. rubens*	*Penicillium*	*Chrysogena*	*Chrysogena*	PDH20-03	*P. chrysogenum*	Misidentified
GCA_023626995	JAMAER01	*P. rubens*	*Penicillium*	*Chrysogena*	*Chrysogena*	PDH20-04	*P. chrysogenum*	Misidentified
GCA_023626795	JAMAEJ01	*P. rubens*	*Penicillium*	*Chrysogena*	*Chrysogena*	PF2508S	*P. chrysogenum*	Misidentified
GCA_023624355	JAMACM01	*P. rubens*	*Penicillium*	*Chrysogena*	*Chrysogena*	PF3401	*P. chrysogenum*	Misidentified
GCA_023626695	JAMAED01	*P. rubens*	*Penicillium*	*Chrysogena*	*Chrysogena*	PH4103	*P. chrysogenum*	Misidentified
GCA_023627055	JAMAEU01	*P. rubens*	*Penicillium*	*Chrysogena*	*Chrysogena*	PK3604	*P. chrysogenum*	Misidentified
GCA_023624415	JAMACJ01	*P. rubens*	*Penicillium*	*Chrysogena*	*Chrysogena*	PL40-01	*P. chrysogenum*	Misidentified
GCA_023624255	JAMACQ01	*P. rubens*	*Penicillium*	*Chrysogena*	*Chrysogena*	PM3404	*P. chrysogenum*	Misidentified
GCA_023626595	JAMADY01	*P. rubens*	*Penicillium*	*Chrysogena*	*Chrysogena*	PYS3203	*P. chrysogenum*	Misidentified
GCA_023624295	JAMACO01	*P. rubens*	*Penicillium*	*Chrysogena*	*Chrysogena*	R1210	*P. chrysogenum*	Misidentified
GCA_023626775	JAMAEI01	*P. rubens*	*Penicillium*	*Chrysogena*	*Chrysogena*	R1211B	*P. chrysogenum*	Misidentified
GCA_023627175	JAMAFB01	*P. rubens*	*Penicillium*	*Chrysogena*	*Chrysogena*	R13B	*P. chrysogenum*	Misidentified
GCA_023626835	JAMAEK01	*P. rubens*	*Penicillium*	*Chrysogena*	*Chrysogena*	R20-04	*P. chrysogenum*	Misidentified
GCA_023627135	JAMAEY01	*P. rubens*	*Penicillium*	*Chrysogena*	*Chrysogena*	R20-05	*P. chrysogenum*	Misidentified
GCA_023624395	JAMACK01	*P. rubens*	*Penicillium*	*Chrysogena*	*Chrysogena*	R20-08	*P. chrysogenum*	Misidentified
GCA_023624165	JAMADX01	*P. rubens*	*Penicillium*	*Chrysogena*	*Chrysogena*	R2501	*P. chrysogenum*	Misidentified
GCA_023624185	JAMACT01	*P. rubens*	*Penicillium*	*Chrysogena*	*Chrysogena*	R3104	*P. chrysogenum*	Misidentified
GCA_023626915	JAMAEN01	*P. rubens*	*Penicillium*	*Chrysogena*	*Chrysogena*	R3301	*P. chrysogenum*	Misidentified
GCA_023627275	JAMAFG01	*P. rubens*	*Penicillium*	*Chrysogena*	*Chrysogena*	R3406	*P. chrysogenum*	Misidentified
GCA_023624275	JAMACR01	*P. rubens*	*Penicillium*	*Chrysogena*	*Chrysogena*	R4101	*P. chrysogenum*	Misidentified
GCA_023624335	JAMACN01	*P. rubens*	*Penicillium*	*Chrysogena*	*Chrysogena*	R4403	*P. chrysogenum*	Misidentified
GCA_023626935	JAMAEO01	*P. rubens*	*Penicillium*	*Chrysogena*	*Chrysogena*	S1301	*P. chrysogenum*	Misidentified
GCA_023626955	JAMAEQ01	*P. rubens*	*Penicillium*	*Chrysogena*	*Chrysogena*	S1302	*P. chrysogenum*	Misidentified
GCA_023627335	JAMAFI01	*P. rubens*	*Penicillium*	*Chrysogena*	*Chrysogena*	S20-02	*P. chrysogenum*	Misidentified
GCA_023626875	JAMAEL01	*P. rubens*	*Penicillium*	*Chrysogena*	*Chrysogena*	S3404	*P. chrysogenum*	Misidentified
GCA_023627095	JAMAEV01	*P. rubens*	*Penicillium*	*Chrysogena*	*Chrysogena*	S3406	*P. chrysogenum*	Misidentified
GCA_023627115	JAMAEX01	*P. rubens*	*Penicillium*	*Chrysogena*	*Chrysogena*	S40-01	*P. chrysogenum*	Misidentified
GCA_019828795	JACVQW01	*P. rubens*	*Penicillium*	*Chrysogena*	*Chrysogena*	SN302OCP1	*P. fimorum*	Misidentified (wrong section)
GCA_019827435	JACVQT01	*P. rubens*	*Penicillium*	*Chrysogena*	*Chrysogena*	SN302OCR3	*P. fimorum*	Misidentified (wrong section)
GCA_023627235	JAMAFE01	*P. rubens*	*Penicillium*	*Chrysogena*	*Chrysogena*	X20-07	*P. chrysogenum*	Misidentified
GCA_023627075	JAMAEW01	*P. rubens*	*Penicillium*	*Chrysogena*	*Chrysogena*	Y1301	*P. chrysogenum*	Misidentified
GCA_025768145	JAMFOS01	*P. rubens*	*Penicillium*	*Chrysogena*	*Chrysogena*	Y3303	*P. chrysogenum*	Misidentified
GCA_023624135	JAMADW01	*P. rubens*	*Penicillium*	*Chrysogena*	*Chrysogena*	Y40-02	*P. chrysogenum*	Misidentified
GCA_028828895	JAQKAT01	*P. rudallense*	*Aspergilloides*	*Aspergilloides*	*Glabra*	IBT35674	*Penicillium* species	Identified to genus
GCA_019775415	JACWFT01	*P. rudallense*	*Aspergilloides*	*Aspergilloides*	*Glabra*	VSIDKN	*Penicillium* species	Identified to genus
GCA_019775465	JACWFS01	*P. rudallense*	*Aspergilloides*	*Aspergilloides*	*Glabra*	VSIDKN3	*Penicillium* species	Identified to genus
GCA_027569595	JAPDFJ01	*P. sanguifluum*	*Aspergilloides*	*Citrina*	*Roseopurpurea*	G339	*Penicillium* species	Identified to genus
GCA_027569895	JAPDLA01	*P. silybi*	*Aspergilloides*	*Exilicaulis*	*Restricta*	G342	*Penicillium* species	Identified to genus
GCA_013138035	JAASRZ01	*P. solitum*	*Penicillium*	*Fasciculata*	*Camembertiorum*	12	*Penicillium* species	Identified to genus
GCA_028828285	JAQJAL01	*P. solitum*	*Penicillium*	*Fasciculata*	*Camembertiorum*	IBT35410	*P. majusculum*	Synonym
GCA_003800495	PUXE02	*P. solitum*	*Penicillium*	*Fasciculata*	*Camembertiorum*	SPGF1	*Penicillium* species	Identified to genus
GCA_019804565	JAETFV01	*P. solitum*	*Penicillium*	*Fasciculata*	*Camembertiorum*	VSIIIDKN3.2	*P. robsamsonii*	Misidentified (wrong section)
GCA_028828935	JAQJZS01	*P. velutinum*	*Aspergilloides*	*Exilicaulis*	*Lapidosa*	IBT18751	*Penicillium* species	Identified to genus
GCA_028828825	JAQJZP01	*P. yarmokense*	*Penicillium*	*Canescentia*	*Canescentia*	IBT19259	*P. canescens*	Misidentified
GCA_028827225	JAPZBW01	*Penicillium* sp. nov. *cyclopium*	*Penicillium*	*Fasciculata*	*Viridicata*	IBT12396	*Penicillium* species	Undescribed species
GCA_028828675	JAQJZK01	*Penicillium* sp. nov. *umkohba*	*Aspergilloides*	*Sclerotiorum*	*Herqueorum*	IBT29812	*P. herquei*	Undescribed species
GCA_011750695	WTTZ01	*Talaromyces minnesotensis*	*–*	*Trachyspermi*	*–*	OUCMDZ-019	*Penicillium* species	Misidentified (Incorrect genus)
GCA_002382855	NPFK01	*Talaromyces pinophilus*	*–*	*Talaromyces*	*–*	CL100	*P. occitanis*	Misidentified (Incorrect genus)
GCA_002382835	NPFJ01	*Talaromyces pinophilus*	*–*	*Talaromyces*	*–*	CT1	*P. occitanis*	Misidentified (Incorrect genus)

There are many reasons why genome sequences may have been submitted with names with which we disagree. The aim of this revision was not to criticise the submitters. Rather, we want to make our opinions known about the species to which available genomes belong, thus making the already very important resource that the submitters have created even more valuable. Based on our re-identifications, *Penicillium* genomes are currently available for 103 of 536 accepted species, representing both subgenera, 22 of 33 sections and 51 of 101 series.

*Authors*: Cobus M. Visagie*, Jens C. Frisvad, and Jos Houbraken.

*Contact: Cobus.Visagie@fabi.up.ac.za.

## IMA GENOME‐F 18B

### Draft genome sequences of *Penicillium *species from dry cured meat, *Penicillium biforme, Penicillium brevicompactum *and* Penicillium solitum*, isolated from Italian salami

#### Introduction

Fungi impact the breadth of biotechnology, animal and plant health, and food production. However, the knowledge of the contribution of molds in seasoned foods is still very limited compared to bacteria and yeasts (Tamang et al. 2016). Some species are used in the production of a particular fermented food, with significant contributions to improving food safety, nutritional value, organoleptic quality and contributing to food preservation (Bourdichon et al. 2012). Conversely, some molds are considered food contaminants causing spoilage and the production of toxic metabolites (EFSA 2023; Davies et al. 2021; Rico-Munoz et al. 2019; Avery et al. 2019).Mold growth is often considered an undesirable characteristic of aged products, with some exceptions, such as the use of *Penicillium camemberti* and *Penicillium roqueforti* in cheeses (Ropars et al. 2020a; Hymery et al. 2014), and *Penicillium nalgiovense* and *Penicillium salamii* on cured meats (Magistà et al. 2016; Mintzlaff and Leistner 1972). Some of the microbial species naturally found on fermented foods today originated from “domestication events”. This process selected for the beneficial traits of wild species in some fermented food production, although this does not necessarily mean that they are suitable for other fermented foods (Bourdichon et al. et al. 2012; Kaur and Dua 2022). Besides *P. nalgiovense* and *P. salamii*, and according to the literature (Bourdichon et al. 2012; Alapont et al. 2014), we often isolated other *Penicillium* species during our investigations of the mold population on cured meat products, such as *Penicillium biforme, P. brevicompactum,* and* P. solitum.*

*Penicillium biforme*, has been hypothesized to have originated from the domestication of wild *P. fuscoglaucum* on cheese, in an episode independent of the one that led to the domestication of the sister species *P. camemberti* (Steenwyk 2019; Ropars et al. 2020b). Growth of *P. biforme* occurs more rapidly on salted than unsalted medium (Ropars et al. 2020b), and it is found on cheese rinds such as Tommes or fresh goat cheeses. It is also used to produce dry-cured meat (Ropars and Giraud 2022). Despite having a functional biosynthetic pathway, the lack of accumulation of ergot alkaloids, such as rugulovasines, in *P. biforme*-aged cheese has been explained as nutrition-driven gene regulation preventing this fungus from producing ergot alkaloids in dairy products (Fabian et al. 2018).

*Penicillium brevicompactum* is often reported as a contaminant in spoiled dairy products (Garnier et al. 2017) and may contribute to the pleasant organoleptic characteristics of cured meats (Parussolo et al. 2019) due to its known lipolytic and proteolytic activity (Alapont et al. 2015). The main drawback to the presence of *P. brevicompactum* on cured meat, concerns the production of mycophenolic acid mycotoxin (Frisvad et al. 2004).

*Penicillium solitum* has been reported as a contaminant of cheese by several authors (Ramos-Pereira et al. 2019; Lund et al. 1995) but it is one of the predominant species in several dry-cured meat products, such as dry-cured sausages (Sørensen et al. 2008; Lopez Dıaz et al. 2001) and speck (Peintner et al. 2000). Although some studies include *P. solitum* among the producers of cyclopiazonic acid mycotoxin (Alapont 2014), it does not produce mycotoxins (Frisvad 2014) and its genome harbors only a partial cluster for the biosynthesis of patulin mycotoxin (Wu et al. 2019). It has been hypothesized that *P. solitum* could be responsible for the phenolic defect of hams, an unpleasant odor known to be caused by *P. commune* contamination (Scaramuzza et al. 2015), however it currently belongs to the list of seven *Penicillium* species reported with beneficial use for foods (Bourdichon et al. 2012).

*Penicillium* was amongst the predominant fungal genus capable of growth on the surface of dry fermented meats (Perrone et al. 2015; Magistà et al. 2017). Here we report the genome of three isolates of *Penicillium* from Italian salami, *P. biforme* ITEM 15300, *P. brevicompactum* ITEM 18316, and *P. solitum* ITEM 18327, respectively, which have been shown to be adapted to the curing conditions of fermented sausages production.

#### Sequenced strains

*Penicillium brevicompactum*: **Italy:** *Puglia*: Martina Franca, isolated from typical sausage “pagnottella in crusca”, 2019, *D. Magistà* (ITEM 18316).

*Penicillium biforme*: **Italy:** *Calabria*: Cosenza, isolated from Italian salami “Capocollo”, 2012, *G. Perrone* (ITEM 15300).

*Penicillium solitum*: **Italy:** *Puglia*: Martina Franca, isolated from typical sausage “pagnottella in crusca”, 2019, *D. Magistà* (ITEM 18327).

#### Nucleotide sequence accession number

The genome assembly and annotations are available from the JGI Fungal Genome Portal MycoCosm (Grigoriev et al. 2014; https://mycocosm.jgi.doe.gov/) under JGI Project ID: 1,289,827, 1,289,819, 1,289,903, for *P. biforme* ITEM 15300, *P. brevicompactum* ITEM 18316, and *P. solitum* ITEM 18327, respectively. The whole-genome shotgun projects were deposited in the NCBI GenBank database under accession numbers: JASJSW000000000 (ITEM 15300) JASJRY000000000 (ITEM 18316), JASJUE000000000 (ITEM 18327), [BioProjects: PRJNA970850 (ITEM 15300), PRJNA971651 (ITEM 18316), PRJNA970851 (ITEM 18327), and BioSamples: SAMN35016899 (ITEM 15300), SAMN35051276 (ITEM 18316), SAMN35016900 (ITEM 18327)].

#### Materials and methods

The fungal isolates of *Penicillium biforme* ITEM 15300, *P. brevicompactum* ITEM 18316 and *P. solitum* ITEM 18327, were deposited as public resources at the Agri-Food Microbial Culture Collection—ITEM (http://server.ispa.cnr.it/ITEM/Collection/). The genomic DNA was extracted from mycelium grown in Potato Dextrose Broth (Oxoid-Thermo Fisher, UK) in the dark at 25 °C, 200 rpm for 5 days. For RNA extraction each strain was inoculated as a spore suspension on Milk (50% v/v) agar, Brain Heart Infusion agar (Oxoid-Thermo Fisher, UK) and Yeast Extract Sucrose agar (Oxoid-Thermo Fisher, UK), and were grown in the dark at 25 °C for 5 days. The mycelia were recovered by vacuum filtration and ground in liquid nitrogen. Genomic DNA was isolated using the DNeasy Plant Mini Kit (Qiagen, Germany), and total RNA was extracted using the RNeasy Plant Mini Kit (Qiagen, Germany), following the manufacturer’s instructions. Quality and integrity of DNA and RNA was checked with NanoDrop 1000 (Thermo Fisher, UK) and Bioanalyzer 2100 (Agilent, Italy) analysis.

The draft genomes of ITEM 18316, ITEM 15300 and ITEM 18327 and the transcriptomic datasets for genome annotation, were generated with Illumina technology. For DNA library preparation, 200 ng of genomic DNA was sheared to 600 bp using a LE220 focused-ultrasonicator (Covaris, USA). The sheared DNA fragments were size selected by double-SPRI using TotalPure NGS beads (Omega Bio-tek, USA) and selected fragments were end-repaired, A-tailed, and ligated with Illumina compatible unique dual-index sequencing adaptors (IDT, USA). Libraries were sequenced on the NovaSeq 6000 (Illumina, USA) sequencing platform using NovaSeq XP v1 reagent kits (Illumina, USA), S4 flow cell, and following a 2 × 150 indexed run.

For the transcriptomic datasets, stranded cDNA libraries were generated using the Illumina Truseq Stranded mRNA Library Prep kit. Equimolar aliquots of RNA from each medium were pooled and used for library preparation. The mRNA fraction was purified from 1 ug of pooled total RNA using magnetic beads containing poly-T oligos, fragmented and reversed transcribed using random hexamers and SSII (Invitrogen, USA) followed by second strand synthesis. The prepared libraries were sequenced on the Illumina NovaSeq 6000 sequencing platform using NovaSeq XP v1 reagent kits (Illumina, USA), S4 flow cell, following a 2 × 150 indexed run.

Raw Illumina reads were filtered by Decontamination Using Kmers (BBDuk) in BBtools v. 38.79 (Bushnell 2020) which is capable of quality-trimming and filtering, adapter-trimming, and contaminant-filtering via kmer matching. Two million of the filtered genomic reads were subsampled to assemble the mitochondrial genome using GetOrganelle v. 1.7.1 (Jin et al. 2020). The nuclear genome assemblies were generated with SPAdes v. 3.14.1 (Bankevich et al. 2012), using a 20.0 M read-pair subsample of the resulting non-organellar reads, obtained by removing any organelle matching reads with BBtools v. 38.79 (Bushnell 2020). The filtered transcriptomic reads were used as input for de novo assembly of RNA contigs using Trinity v. 2.11.0 (Grabherr et al. 2011).

The genome assemblies were masked for repeats using RepeatMasker (Smit et al*.*^1996^–2010) with the RepBase library 25.03 (Bao et al. 2015) and the most frequent repeats were identified by RepeatScout (Price et al. 2005). The completeness of the genome assemblies was performed with Benchmarking Universal Single-Copy Orthologs (BUSCO) v. 5.4.6, implemented in the Galaxy platform, using the eurotiales_odb10 lineage dataset (Manni et al. 2021). The nuclear genome was annotated using the JGI Annotation pipeline (Grigoriev et al. 2014) using a combination of ab initio, homology-based, and transcriptome-based gene models predicted from assembled RNA-sequencing data derived from the cultured fungus.

Predicted proteins were functionally annotated using SignalP v. 3 (Nielsen et al. 1997), TMHMM v. 2.0 (Melén et al. 2003), InterProScan v. 5.9–50.0 (Quevillon et al. 2005), and BLASTp alignments against the NCBI NR, SwissProt, KEGG (Kanehisa et al. 2006), and KOG (Koonin et al. 2004) databases. Transcription factors were assigned based on Pfam domains. Gene ontology (GO) terms (Ashburner et al. 2000) were assigned based on InterPro and SwissProt hits. Protein alignments by BLASTp against TCDB were used for transporter classifications (Saier et al. 2016) and MEROPS for peptidase classifications (Rawlings et al. 2014). CAZymes were annotated as described in Lombard et al. (2014). Secondary metabolite clusters and classifications were inferred from Pfam domain content and physical proximity based on the SMURF algorithm (Khaldi et al. 2010). Cytochrome 450 (CYP) subfamilies were assigned based on HMMs, which were labelled based on homology to manually curated CYP genes (Nelson 2009). The HMMs corresponding to different CYP subfamilies were derived from CYP sequences in NCBI NR and MycoCosm (~ 220 K sequences) followed by UCLUST clustering (Edgar 2010) and iterative HMM building. Finally, biosynthetic gene clusters (BGCs) were identified using anti-SMASH fungal v. 6.1.1 (Blin et al. 2021) with default parameters.

The taxonomic identity of the species was confirmed by phylogenetic analysis of four combined gene regions. Partial DNA sequences from the internal transcribed spacer (ITS) region, calmodulin (*CaM*) gene, beta-tubulin (*BenA*) gene, and DNA-dependent RNA polymerase II second largest subunit (*RPB2*) gene, were extracted from the genomes. The nucleotide sequences of *Penicillium* type strains in sections *Brevicompacta* and *Fasciculata* were retrieved from GenBank, following Houbraken et al. (2020). The sequences were aligned using the online version of MAFFT v. 7. (Katoh et al*.* 2019). IQ-TREE v. 2.2.0 (Minh et al. 2020) implemented ModelFinder (Kalyaanamoorthy et al. 2017) to calculate the best-fit model according to the Bayesian Information Criterion (BIC) score on the partitioned dataset (Chernomor et al. 2016), and infer the Maximum Likelihood phylogenetic tree based on 10,000 ultrafast bootstrap support (Hoang et al. 2018).

#### Results and discussion

The draft genomes of *Penicillium brevicompactum* ITEM 18316, *P. biforme* ITEM 15300, and *P. solitum* ITEM 18327 were generated at the DOE Joint Genome Institute (JGI) using Illumina short-read sequencing. A total of 63,689,624 (*P. brevicompactum* ITEM 18316), 50,226,000 (*P. biforme* ITEM 15300) and 38,958,788 (*P. solitum* ITEM 18327) raw genomic reads were generated, yielding after filtering 62,681,478 (9.3974 Gb), 49,558,002 (7.4284 Gb) and 38,398,144 (5.7561 Gb) reads, respectively. A total of 173,983,720 (*P. brevicompactum* ITEM 18316), 207,916,384 (*P. biforme* ITEM 15300) and 112,809,614 (*P. solitum* ITEM 18327) raw transcriptomic reads were generated of which 22.4%, 22.1% and 8.8% were discarded during filtering, respectively. The draft genome assembly process resulted in 156 scaffolds for *P. brevicompactum* ITEM 18316, 473 scaffolds for *P. biforme* ITEM 15300, and 409 scaffolds for *P. solitum* ITEM 18327 with total consensus genome size of 31.00 Mb, 35.45 Mb and 34.18 Mb, respectively (Table 2). Additionally, one contig was generated for mitochondrial genome with a length of 29.93 Kb, 28.02 Kb and 28.23 Kb for *P. brevicompactum* ITEM 18316, *P. biforme* ITEM 15300, and *P. solitum* ITEM 18327, respectively. Statistics regarding the completeness of genome assemblies performed with BUSCO are shown in Table 3. The gene prediction revealed that *P. brevicompactum* ITEM 18316, *P. biforme* ITEM 15300 and *P. solitum* ITEM 18327 harbor 11,719, 12,781 and 12,589 protein coding genes, respectively. The genome size and number of predicted proteins of *P. brevicompactum* ITEM 18316 and *P. solitum* ITEM 18327 are comparable with the average of the three isolates of *P. brevicompactum* and two isolates of *P. solitum* recently compared by Petersen et al. (2023). Details of coding genes, exons and introns of *P. brevicompactum* ITEM 18316, *P. biforme* ITEM 15300 and *P. solitum* ITEM 18327 genomes are shown in Table 4.

Annotation of secondary metabolites performed with anti-SMASH confirmed the presence of the known gene cluster for mycophenolic acid mycotoxin biosynthesis in *P. brevicompactum* ITEM 18316, while it highlighted the presence of the gene cluster for PR-toxin biosynthesis in *P. biforme* ITEM 15300, a mycotoxin never reported in *P. biforme* (Houbraken et al. 2016). PR-toxin was first characterized in *P. roqueforti*, but due to its low stability in cheese it has rarely been a cause for concern (Bourdichon et al. 2012). No known mycotoxin-producing biosynthetic gene clusters were found in *P. solitum* ITEM 18327.

**Fig. 3:**
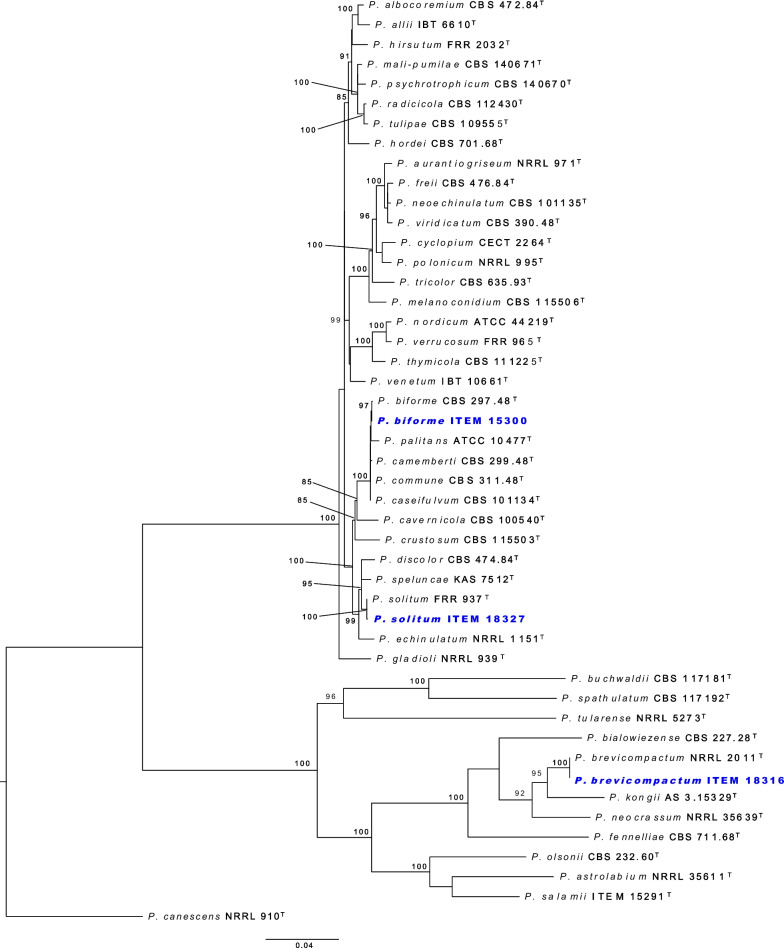
ML phylogenetic tree of *Penicillium* sections *Fasciculata* and *Brevicompacta* indicating the three sequenced isolates (in blue), and ultrafast bootstrap support at the nodes.

Phylogenetic analysis confirmed the identity of the three strains (Fig. 3). *Penicillium brevicompactum* ITEM 18316 and *P. solitum* ITEM 18327 were assigned to the respective species, also *P. biforme* ITEM 15300 appears to be well distinguished from sister species in series *Camembertiorum*. Studies argue that the taxonomy of the *camemberti* clade has not been fully resolved with potent genetic markers, thus several misidentified isolates have recently been reassigned to the right species using whole-genome-based analyses (Ropars et al. 2020b). The *P. biforme* ITEM 15300 clusters with the type species of *P. biforme* with high bootstrap support. The availability of these three genomes of *Penicillium* species from dry-cured meat environments will allow for new comparative studies for species adapted to this challenging but economically important environment.

*Authors*: Donato Magistà*, Massimo Ferrara, Maria De Angelis, Yu Zhang, Emily Savage, Sara Calhoun, Richard D. Hayes, Jasmyn Pangilinan, Kurt LaButti, Anna Lipzen, Vivian Ng^**3**^** Igor V. Grigoriev, Scott E. Baker, and Giancarlo Perrone.**

*Contact: donato.magista@ispa.cnr.it.

**Table 2:** Whole genome assembly features of the three *Penicillium* species isolated from Italian salami.

Genome Assembly	*P. brevicompactum* **ITEM 18316**	*P. biforme* **ITEM 15300**	*P. solitum* **ITEM 18327**
Genome Assembly size (Mbp)	31.00	35.45	34.18
Sequencing read coverage depth	191.88x	178.91x	168.42x
Contigs	212	532	454
Scaffolds	156	473	409
Scaffolds > = 2Kbp	122	377	351
Scaffold N50	6	24	26
Scaffold L50 (Mbp)	2.82	0.47	0.42
Contigs N50	14	37	46
Contigs L50 (Mbp)	0.67	0.30	0.20
Scaffold max	3,287,503	1,455,359	1,444,804
Contigs max	3,131,022	1,143,950	793,477
GC (%)	49.62	47.78	48.23

**Table 3:** Completeness of the genome assemblies of the three *Penicillium* species isolated from Italian salami calculated with BUSCO.

**BUSCO completeness statistics**	*P. brevicompactum* **ITEM 18316**	*P. biforme* **ITEM 15300**	*P. solitum* **ITEM 18327**
BUSCO overall completeness %	98.9	100.0	99.9
Single copy BUSCOs	4132	4182	4174
Duplicated BUSCOs	12	8	13
Fragmented BUSCOs	0	0	0
Missing BUSCOs	47	1	4
Total BUSCO groups searched	4191	4191	4191

**Table 4:** Details of coding genes, exons, and introns of the three *Penicillium* species isolated from Italian salami.

Gene Models	*P. brevicompactum* ITEM 18316		*P. biforme* ITEM 15300		*P. solitum* ITEM 18327	
Length (bp) of:	**Average**	**Median**	**Average**	**Median**	**Average**	**Median**
Gene	1956	1705	1942	1670	1941	1675
Transcript	1798	1566	1764	1513	1760	1520
Exon	574	347	552	330	548	328
Intron	76	58	83	61	84	61
Protein length (aa)	483	399	480	395	480	394
Exons per gene	3.13	3	3.19	3	3.21	3

## IMA GENOME‐F 18C

### Draft genome sequence of *Penicillium cvjetkovicii* isolated from Italian salami

#### Introduction

The ascomycete genus *Penicillium* is home to tremendous biochemical and enzymatic diversity that contributes to its phenotype and may also have use in biotechnology. *Penicillium cvjetkovicii* belongs to the slow-growing subgenus *Aspergilloides*, section *Cinnamopurpurea*, series *Idahoensia* (Houbraken et al. 2020). Species from this section have been found as contaminants on food products, such as *P. fluviserpens* isolated from tomato fruit (Nguyen et al. 2020), *P. cinnamopurpureum* and *P. malacaense* reported from meju samples in Korea (Jung et al. 2012; Kim et al. 2015), and *P. cinnamopurpureum* contributing to the potato-taste-defect of coffee (Hale et al. 2022).

*Penicillium cvjetkovicii* has been described relatively recently (Peterson et al. 2015). A cheese isolate was originally obtained from Spain (Marin et al. 2014) and identified by Peterson et al. (2015) as *P. cvjetkovicii*, although it had initially been considered to be *P. chermesinum* (Marin et al. 2014, Peterson et al. 2015). Morphologically, this fungus is characterized by the monoverticillata penicilli and the production of vinaceous to reddish-brown soluble pigments. The production of the vinaceous to reddish-brown soluble pigments, which is typically observed in most of the species belonging to section *Cinnamopurpurea* (Peterson et al. 2015), was probably related to the observation of small dark spots on the surface of aged cheeses (Ramos-Pereira et al. 2019). Two species in this section, *P. colei* and *P. monsserratidens* produce citreoviridin, a mycotoxin synthesized by highly reducing polyketide synthases. Similar to other *Penicillium* species in this section, however, *P. cvjetkovicii* is not known to produce mycotoxins or other toxic metabolites (Peterson et al. 2015). Other species in section *Cinnamopurpurea* are hypothesized to be beneficial in developing the unique characteristics of typical foods, such as, *P. gravinicasei* recently isolated from cheese (Anelli et al. 2018), and *P. nodulum* which participates in the softening of cassava tissue during the fermentation of cassava dough into agbelima (Amoa-Awua 1997).

Very few species belonging to other sections are associated with cheese. This is the case of *P. glabrum *(section *Aspergilloides*), the recently described *P. cvjetkovicii* (section *Cinnamopurpurea*) and *P. citrinum* (section *Citrina*) (Houbraken et al. 2020).

Here we report the genome of an isolate of *P. cvjetkovicii*, which was isolated from a dry cured sausage and tested as a surface mold starter to produce the same product.

#### Sequenced strain

**Italy:** *Puglia*: Martina Franca, Taranto, isolated from Italian salami, 2019, *D. Magistà* (ITEM 18317).

#### Nucleotide sequence accession number

The genome assembly and annotations are available from the JGI Fungal Genome Portal MycoCosm (Grigoriev et al. 2014; https://mycocosm.jgi.doe.gov/) under JGI Project ID 1289847 and has been deposited at GenBank under BioProject number PRJNA971650(BioSample n. SAMN35051277; Project Accession n. SRP442271).

#### Materials and methods

*Penicillium cvjetkovicii* ITEM 18317 is deposited and available at the International ITEM culture collection (CNR-ISPA, Bari, Italy—http://www.ispa.cnr.it/Collection/).

For genomic DNA extraction, the mycelium was grown in potato dextrose broth (PDB) in the dark at 25 °C, 200 rpm for 5 d. The mycelium was recovered by vacuum filtration and ground in liquid nitrogen. The DNeasy Plant Mini Kit (Qiagen, Germany), was used to extract genomic DNA following the manufactures’ instructions. For RNA extraction, the strain was inoculated as a spore suspension on milk (50% v/v) agar, brain heart infusion agar (Oxoid, UK) and yeast extract sucrose (YES) agar and grown in the dark at 25 °C for 5 days. The mycelia was collected, ground in liquid nitrogen and total RNA was extracted using the RNeasy Plant Mini Kit (Qiagen, Germany), according to the manufactures’ instructions. Quality and integrity of DNA and RNA was checked with NanoDrop and Bioanalyzer 2100 analysis.

The draft genome of *P. cvjetkovicii* ITEM 18317 and the transcriptomic datasets for genome annotation were generated with Illumina technology. For DNA library preparation, genomic DNA was sheared using a Covaris LE220 focused-ultrasonicator. The sheared DNA fragments were used for library preparation and sequenced on the Illumina NovaSeq 6000 sequencing platform 2 × 150 indexed run. Raw Illumina reads were filtered for quality and artifacts using BBTools software package v. 38.79 (Bushnell 2020). Mitochondrial genome was assembled using get_organelle v1.7.1 (Jin et al*.* 2018). The genome assembly was generated with SPAdes v3.14.1 (Bankevich et al. 2012) filtering out the resulting organellar reads. For the transcriptomic dataset, stranded cDNA libraries were generated and sequenced on the Illumina NovaSeq 6000 sequencing platform 2 × 150 indexed run. The raw Illumina reads were filtered and trimmed for quality and artifacts using BBTools software package v. 38.79 (Bushnell 2020) and used as input for de novo assembly of RNA contigs using Trinity (v2.11.0) (Grabherr et al. 2011).

The genome assembly was masked for repeats using RepeatMasker (Smit et al.^1996^–2010) with the RepBase library v25.03 (Jurka et al. 2005) and the most frequent repeats were identified by RepeatScout (Price et al. 2005). The genome assembly completeness was estimated with BUSCO v5.4.6 (lineage dataset: eurotiales_odb10), implemented in the Galaxy platform (Manni et al. 2021). The nuclear genome was annotated with the JGI Annotation pipeline (Grigoriev et al. 2014), using a combination of ab initio, homology-based, and transcriptome-based gene predictors. Predicted proteins were functionally annotated using SignalP v3 (Nielsen et al.^1997^), TMHMM v2.0 (Melén et al. 2003), InterProScan 5.9–50.0 (Quevillon et al. 2005), and BLASTp alignments against the NCBI NR, SwissProt, KEGG (Kanehisa et al. 2006), and KOG (Koonin et al. 2004) databases. Transcription factors were assigned based on Pfam domains. Gene ontology (GO) terms (Ashburner et al. 2000) were assigned based on InterPro and SwissProt hits. Protein alignments by BLASTp against TCDB were used for transporter classifications (Saier et al. 2016) and MEROPS for peptidase classifications (Rawlings et al. 2014). CAZymes were annotated as described in Lombard et al. (2014). Secondary metabolite clusters and classifications were inferred from Pfam domain content and physical proximity based on the SMURF algorithm (Khaldi et al. 2010). Cytochrome 450 (CYP) subfamilies were assigned based on HMMs, which were labelled based on homology to manually curated *CYP* genes (Nelson 2009). The HMMs corresponding to different *CYP* subfamilies were derived from *CYP* sequences in NCBI NR and MycoCosm (~ 220 K sequences) followed by UCLUST clustering (Edgar 2010) and iterative HMM building. Finally, biosynthetic gene clusters (BGCs) were identified using anti-SMASH fungal v. 6.1.1 (Blin et al. 2021) with default parameters.

The taxonomic identity of *P. cvjetkovicii* ITEM 18317 was confirmed with phylogenetic analysis of three combined gene regions. Partial DNA sequences from the internal transcribed spacer (ITS) region, calmodulin (*CaM*) gene, and beta-tubulin (*BenA*) gene, were extracted from the genome. The nucleotide sequences of *Penicillium* ex-type strains in the same section *Cinnamopurpurea* were retrieved from GenBank, following Houbraken et al. (2020). The sequences were aligned using the online version of MAFFT v. 7. (Katoh et al. 2019). IQ-TREE 2.2.0 (Minh et al. 2020), implemented with ModelFinder (Kalyaanamoorthy et al. 2017), was used to calculate the best-fit model according to the Bayesian Information Criterion (BIC) score on the partitioned dataset (Chernomor et al. 2016), and infer the Maximum Likelihood phylogenetic tree based on 10,000 ultrafast bootstrap support (Hoang et al. 2018).

#### Results and discussion

Fermented meat products represent a significant part of the Mediterranean diet and their production and commercialization contribute to the local economies (Baka et al. 2011). In the last years, many studies have focused on the characterization of fermented sausages microbiota (Ferrocino et al. 2018; Magistà et al. 2016; Perrone et al. 2015). Many fungal species are well adapted to the ecological conditions encountered during the meat fermentation. That is why many fermented meat products, including fermented sausages, are characterized by yeasts and molds growth on the casing surface. The mycobiota of traditional dry-cured meat products is usually characterized by the presence of mould species belonging to the *Penicillium* genus, mainly *P. solitum*, *P. nalgiovense*, *P. chrysogenum*, *P. olsonii*, *P. commune,* and *P. salamii* (Magistà et al. 2017). Besides *P. nalgiovense*, other mould species might be isolated from fermented meat products, probably transferred to the casing surface through airborne contamination or through the use of spices or salt. This is the case of *P. cvjetkovicii* ITEM 18317 isolated from an Italian salami. The draft genome of strain ITEM 18317 was generated at the DOE Joint Genome Institute (JGI) using Illumina short read sequencing technology. A total of 52,369,604 raw reads were generated, yielding 51,612,264 reads (7.74 Gb) after filtering. For transcriptome sequencing, 180,428,788 reads were generated, of which about 30% were discarded after filtering.

**Fig. 4:**
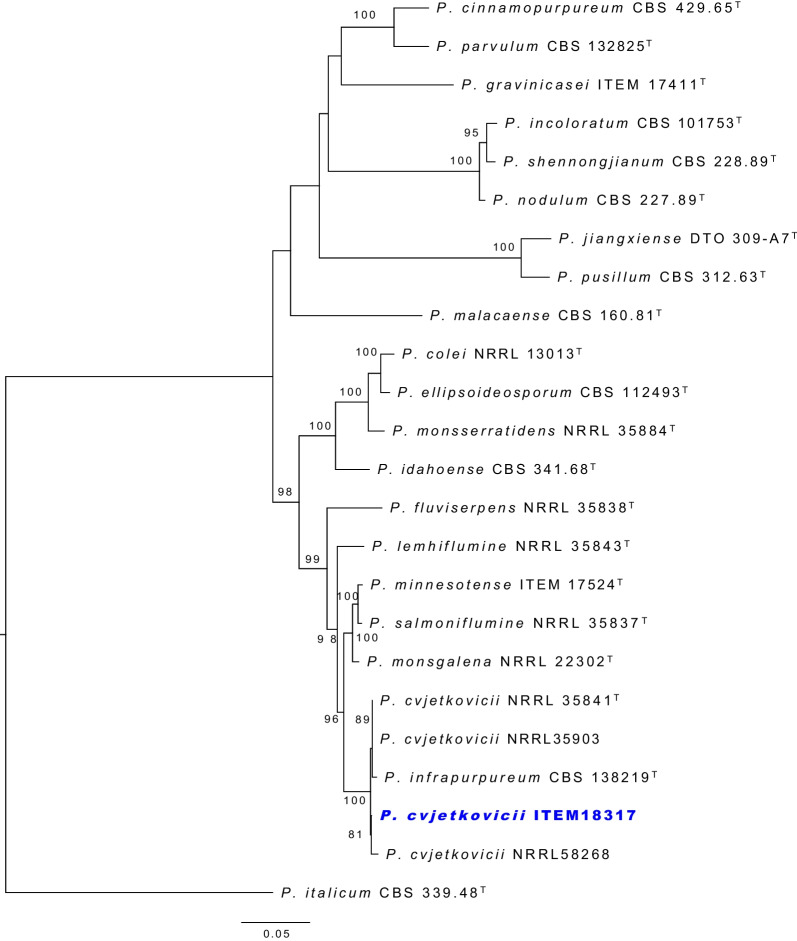
ML phylogenetic tree of *Penicillium* section *Cinnamopurpurea,* indicating the sequenced isolate of *P. cvjetkovicii* ITEM 18317 (in blue), and ultrafast bootstrap support at the nodes.

The draft genome assembly process yielded 272 contigs and 202 scaffolds with a genome size of 25.62 Mbp (Table 5). Additionally, one contig was generated for the mitochondrial genome with a length of 27.19 Kbp. Phylogenetic analysis confirmed the identity of the strain *P. cvjetkovicii* ITEM 18317 (Fig. 4).

Gene prediction produced 9657 protein-coding gene models, of which 7529 were annotated with InterPro (Table 6). Annotation of secondary metabolites performed with anti-SMASH confirmed the absence of mycotoxin-producing biosynthetic gene clusters in *P. solitum* ITEM 18327. Here we present the first draft genome of *P. cvjetkovicii*. Its availability for the scientific community will facilitate the investigation of the biology of this fungus, given our hypothesized application as a fungal starter for meat fermentation.

*Authors*: Massimo Ferrara*, Donato Magistà, Maria De Angelis, Yu Zhang, Emily Savage, Jasmyn Pangilinan, Anna Lipzen, Richard D. Hayes, Vivian Ng, Igor V. Grigoriev, Scott E. Baker, and Giancarlo Perrone.

*Contact: massimo.ferrara@ispa.cnr.it.

**Table 5:** Whole genome assembly features of *Penicillium cvjetkovicii* ITEM 18317.

**Genome Assembly**	
Genome Assembly size (Mbp)	25.62
Sequencing read coverage depth	227.08x
Contigs	212
Scaffolds	202
Scaffolds > = 2Kbp	150
Scaffold N50	10
Scaffold L50 (Mbp)	0.89
Contigs N50	19
Contigs L50 (Mbp)	0.44
Scaffold max	3,152,167
Contigs max	1,324,426
GC (%)	51.26
*BUSCO completeness statistics*	
BUSCO overall completeness %	98.6
Single copy BUSCOs	4125
Duplicated BUSCOs	9
Fragmented BUSCOs	1
Missing BUSCOs	56
Total BUSCO groups searched	4191

**Table 6:** Details of coding genes, exons and introns in *P. cvjetkovicii* ITEM 18317 genome.

**Gene Models**		
***length (bp) of:***	***Average***	***Median***
Gene	1979	1717
Transcript	1820	1586
Exon	577	351
Intron	76	59
Protein length (aa)	486	401
Exons per gene	3.15	3

## IMA GENOME‐F 18D

### Draft genome assemblies of four *Pewenomyces* species from Chile

#### Introduction

*Pewenomyces* is a recently described genus in *Coryneliaceae* (*Eurotiomycetes*, *Coryneliales*) that contains four species: *Pew. kutranfy*, *Pew. lalenivora*, *Pew. tapulicola,* and *Pew. kalosus* (Balocchi et al. 2021, 2022). All these fungi were discovered on *Araucaria araucana* trees in Chile, associated with cankers on branches and young stems. *Pewenomyces kutranfy*, the type species for the genus, was confirmed to be pathogenic and the causal agent of the cankers observed in these trees (Balocchi et al. 2021). The lifestyle of the other three species remains uncertain, but they are most likely saprophytes and/or endophytes (Balocchi et al. 2022). The origin of these *Pewenomyces* species is unknown, although it has been suggested that they are native to the Chilean mountain ranges where they were discovered (Balocchi et al. 2022). This suggeston was based on their temperature preference for growth, the unique and harsh niche where they are found, the species diversity associated with a single host species, and the diversity of *Coryneliaceae* found in Chilean native forests (Fitzpatrick 1942; Butin 1970; Benny et al. 1985a, 1985b; Balocchi et al. 2022). Determining the origin and diversity of these fungi, which is particularly relevant for the emergent pathogen *Pew. kutranfy*, would require research-based evidence. Studies of this nature have not been performed for any other members of the *Coryneliaceae*.

The *Coryneliaceae* is a relatively small fungal family containing approximately 60 species distributed in nine accepted genera. The majority of the species were described before DNA-based techniques were routinely used for taxonomy (Fitzpatrick 1920), and the identity of more than half of the accepted species (including three whole genera) still need to be validated using phylogenetic analyses. Some of the most well-known species in the family are tree pathogens, including *Caliciopsis pinea* on *Pinus* spp. in the USA (Munck et al. 2015), *Hypsotheca pleomorpha* on *Eucalyptus* spp. in Australia (Pascoe et al. 2018), and *Corynelia* spp. on *Podocarpaceae* in South Africa (Wood et al. 2016). Similar to *Pewenomyces* spp., these fungi are mostly assumed to be native where they have been found, a premise based on their occurrence on tree species native to the area and/or apparently narrow geographical distributions (Wood et al. 2016; Pascoe et al. 2018; Migliorini et al. 2020). However, no studies have considered the diversity or biology of these fungi using DNA-based techniques. The genomes of two species of the family, *C. pinea* and *C. orientalis*, are available in open collections (e.g. JGI, GenBank), but no published studies have emerged from these resources. Sequencing the genomes of species in *Pewenomyces* creates further opportunities for studying the biology and evolution of fungi in this relatively small and unique group of fungi, which occur in unique niches distributed around the world.

#### Sequenced strains

*Pewenomyces kutranfy*: **Chile:** *Araucanía (IX), Villarrica National Park sector Puesco*: isolated from cankers on branches of *Araucaria araucana*, 2017, *F. Balocchi* (ex-holotype culture CMW54240 = CBS 146709 = AR128; PREM 63075−dried culture).

*Pewenomyces lalenivora*: **Chile:** *Araucanía (IX), Conguillío National Park, sector Los Paraguas*, spermogonia and ascomata on mature cankers on branches of *Araucaria araucana*, 2019, *F. Balocchi* (ex-type culture CMW56868 = CBS 149332 = FB009; PREM 63252−holotype). *Ralco Natural Reserve*: isolated from cankers on branches of *Araucaria araucana*, 2017, *F. Balocchi* (CMW54250 = CBS 149331 = AR217; PREM 63254−dried culture).

*Pewenomyces tapulicola*: **Chile:** *Biobío (VIII), Nahuelbuta mountain range, Trongol Alto*: isolated from leaves of *Araucaria araucana*, 2017, *F. Balocchi* (CMW54252 = CBS 149335 = AR305; PREM 63251−dried culture).

*Pewenomyces kalosus*: **Chile:** *Araucanía (IX), Conguillío National Park sector Los Paraguas*: isolated from cankers on branches of *Araucaria araucana*, 2017, *F. Balocchi* (ex-type culture CMW54228 = CBS 149329 = AR040; PREM 63245−holotype). *Biobío (VIII): Ralco National Reserve*: isolated from cankers on branches on *Araucaria araucana*, 2017, *F. Balocchi* (CMW56867 = CBS 149328 = AR244; PREM 63246−dried culture).

#### Nucleotide sequence accession number

The genome sequences of *Pewenomyces* spp. have been deposited in DDBJ/EMBL/GenBank databases under the following accession numbers: *Pewenomyces kutranfy* CMW54240 = JAUPWU000000000; *Pew. lalenivora* CMW54250 and CMW56868 = JAUPWS000000000 and JAUPWT000000000 respectively; *Pew. tapulicola* CMW54252 = JAUPWR000000000; and *Pew. kalosus* CMW54228 and CMW 56867 = JAUPWQ000000000 and JAUPWP000000000 respectively.

#### Materials and methods

Cultures of all isolates were obtained from the culture collection (CMW) of the Forestry and Agricultural Biotechnology Institute (FABI), University of Pretoria, South Africa. Isolates were grown in 25 mL glass vials containing ~10 mL of liquid YM broth media (2% malt extract and 0.2% yeast extract) for 4–7 d in the dark with continuous shaking. Mycelia were collected into 2 mL Eppendorf tubes by straining it through sterile gauze, freeze dried, and then ground with metal beads using a mixer mill (MM 301, Retsch GmbH; 30 oscillations/s for 3 min). DNA was extracted using a salt-extraction protocol described by Aljanabi and Martinez (1997) and modified by Duong et al. (2013). Resulting DNA quality and quantity was assessed by gel electrophoresis (1% agarose gel; 12 min at 110 V) and with a Qubit 4 fluorometer (Qubit Assays, Termo Fischer). Illumina whole genome sequencing was carried out by Macrogen where a library with a 350 bp insert size was prepared and sequenced on the NovaSeq 6000 platform to generate 151 bp paired-end reads.

The resulting Illumina reads were trimmed and assembled using CLC Genomics Workbench v 22.0.4 (QIAGEN, Aarhus) with default parameters, discarding contigs shorter than 500 bp. The completeness of the genome was assessed using Benchmarking Universal Single-Copy Orthologs (BUSCO) v. 5.3.2 with the eurotiomycetes_odb10 dataset (Manni et al. 2021). Genome metrics including N50, L50, GC content, and the genome size were obtained using QUAST v 5.0.2 (Mikheenko et al. 2018). The number of protein-coding genes in each genome was predicted using AUGUSTUS v. 3.4.0 (Keller et al. 2011) based on the gene models of *Aspergillus fumigatus*.

The identity of the isolates sequenced in this study were verified by conducting phylogenetic analyses with sequences of the ITS, nc LSU rDNA and *RPB2* gene regions extracted from the resulting assemblies. These analyses were performed by including these sequences in modified datasets obtained from Balocchi et al. (2022), which included representative sequences of most genera in the *Coryneliaceae*. The datasets for each gene region were compiled and aligned using MAFFT (Katoh et al. 2017). Assembled datasets were edited and concatenated using MEGA XI (Tamura et al. 2021) and maximum-likelihood trees were built for the concatenated datasets using the IQ-TREE Web server (Trifinopoulos et al. 2016). Evolutionary models for the analyses were selected using ModelFinder (Kalyaanamoorthy et al. 2017) and statistical support was calculated with Ultrafast Bootstrap analysis (Minh et al. 2013). Resulting trees were visualized and edited using FigTree v.1.4.4 (http://tree.bio.ed.ac.uk/software/figtree/) and edited using Affinity Designer v.1.10.5.1342 (Serif, Nottingham, UK).

#### Results and discussion

**Fig. 5:**
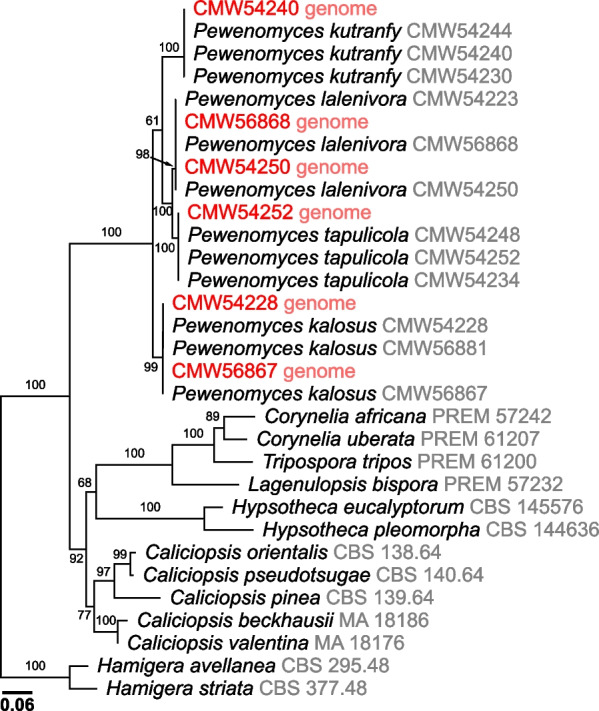
Maximum likelihood tree for the concatenated ITS, nc LSU rDNA and *RPB2 *for members of the *Coryneliaceae*. Sequences extracted from genomes produced in this study are highlighted in red. Numbers on branches indicate Bootstrap values (n = 1000).

Illumina sequencing resulted in 33–39 million reads per isolate, of which between 99.4% and 99.7% remained after trimming. After assembly and filtering, the number of scaffolds per genome varied between isolates of the different species (Table 7). The lowest number of scaffolds was obtained for *Pew. kutranfy* CMW54240 [n = 203; N_50_ = 510 kb], and the largest number of scaffolds were for *Pew. lalenivora* isolates CMW54250 [n = 1,287; N_50_ = 475 kb] and CMW56868 [ n = 2,234; N_50_ = 406 kb]. A similar trend was observed for genome size (Table 7), where *Pew. kutranfy* (CMW54240) had the smallest genome size (29.59 Mb; 8,097 genes), while the two isolates of *Pew. lalenivora* (CMW54250 and CMW56868) had the largest genomes (30.37 Mb; 9,284 genes and 31.66 Mb; 10,432 genes, respectively). Genome coverage ranged between 157× and 186× for all genomes (Table 7). BUSCO completeness scores of the assembled genomes ranged from 91.9% to 92.7%, with the highest values obtained for isolates of *Pew. kutranfy* and *Pew. lalenivora*. Sequences for the ITS, nc LSU rDNA and *RPB2* gene regions extracted from the genomes were identical to those previously obtained by Sanger Sequencing (Balocchi et al. 2021, 2022). Phylogenetic analyses with the individual and concatenated datasets consistently resolved the genome sequenced taxa in the *Pewenomyces* clade next to their corresponding Sanger-based equivalent (Fig. 5), confirming the identity of the sequenced isolates. The genomes produced in this study will provide a valuable future resource for comparative studies involving species in* Pewenomyces* and* Coryneliaceae*. This includes studies ranging from phylogenomics to genome comparisons for biological and ecological questions, mating type system identification, and the development of molecular tools such as microsatellite markers for population genetic studies.

*Authors:* Felipe Balocchi*, Irene Barnes, Brenda D. Wingfield, Anja Piso, Tuan A. Duong*

*Contact: Tuan.Duong@fabi.up.ac.za; felipe.balocchi@fabi.up.ac.za.

**Table 7:** Genome assembly statistics for six isolates representing four *Pewenomyces* species.

Species	Isolate	No of scaffolds	N50 (bp)	L50	GC (%)	Genome size (Mb)	Gene coverage	No of genes (Augustus)	BUSCO [eurotiomycetes_odb10; n = 3546]
*Pewenomyces kutranfy*	CMW54240	203	510,941	16	49.08	29.59	183X	8,097	92.6%[S:92.5%,D:0.1%], F:1.5%, M:5.9%
*Pew. lalenivora*	CMW54250	1,287	475,244	23	50.15	30.37	184X	9,284	92.6%[S:92.5%,D:0.1%], F:1.5%, M:5.9%
	CMW56868	2,328	406,552	25	50.32	31.66	157X	10,432	92.7%[S:92.6%,D:0.1%], F:1.6%, M:5.7%
*Pew. tapulicola*	CMW54252	1,097	661,684	16	50.15	30.49	186X	9,157	92.1%[S:92.0%,D:0.1%], F:1.8%, M:6.1%
*Pew. kalosus*	CMW54228	783	207,244	43	49.38	29.74	180X	8,266	92.1%[S:92.0%,D:0.1%], F:1.7%, M:6.2%
	CMW56867	1,002	185,395	47	49.41	29.78	170X	8,260	91.9%[S:91.8%,D:0.1%], F:1.7%, M:6.4%

## IMA GENOME‐F 18E

### Draft genome sequence of the newly described *Teratosphaeria carnegiei*, associated with *Eucalyptus* leaf spots

#### Introduction

Teratosphaeria leaf blight (TLB) is a collective name used for disease symptoms caused by leaf-infecting *Teratosphaeria* species (*Dothideomycetes, Mycosphaerellales*; Andjic et al. 2019). *Teratosphaeria* species can be found in asymptomatic *Eucalyptus* trees (Kemler et al. 2013; Marsberg et al. 2014), and some only cause mild disease symptoms (Hunter et al. 2011). In contrast, a group of closely related *Teratosphaeria* species with *Kirramyces* asexual morphs are aggressive pathogens and result in severe TLB disease on *Eucalyptus* trees established in plantations, predominantly in areas having tropical and subtropical climates (Andjic et al. 2019). These include species such as *T. destructans*, *T. eucalypti* and *T. pseudoeucalypti*. Most recently, *T. carnegiei* has been described residing in this group of cryptic species (Crous et al. 2022).

*Teratosphaeria carnegiei* was discovered amongst a collection of isolates thought to be those of *T. pseudoeucalypti* isolated from TLB symptoms in a *Eucalyptus grandis* x *E. camaldulensis* plantation in New South Wales (NSW), Australia (Aylward et al. 2021). The isolates were assessed using a microsatellite panel designed to identify TLB species (Havenga et al. 2020) and most isolates were identified as *T. pseudoeucalypti*. However, two of these isolates had genotypes distinct from those of any other TLB pathogens. Phylogenetic analyses showed that these two isolates resided in a monophyletic group with other isolates that had previously been recognized as variants of *T. eucalypti* (Andjic et al. 2010; Crous et al. 2022), but were distinct from both *T. eucalypti* and *T. pseudoeucalypti*.

*Teratosphaeria carnegiei* appears to be of minor economic significance as a pathogen. It has been discovered only twice, both times in northern NSW as part of population-level isolations of *T. eucalypti* or *T. pseudoeucalypti* (Andjic et al. 2010; Crous et al. 2022). It’s low frequency of isolation and co-occurrence with aggressive pathogens raises the question as to whether it can cause disease independently. However, its position as the species most closely related to two damaging TLB pathogens, makes it of considerable interest. This prompted the present study to sequence the genome *T. carnegiei* in order to compare it with other species causing severe TLB.

#### Sequenced strain

**Australia**: New South Wales: isolated from leaf spots on a *Eucalyptus grandis* x *E. camaldulensis* hybrid, 2018, *A.J. Carnegie* (CMW 52470 = PPRI 29908—culture, PREM 63267—dried culture).

#### Nucleotide accession number

The genomic sequences of *T. carnegiei* have been deposited at DDJ/EMBL/GenBank under the accession JANYMD000000000. This paper describes the first version.

#### Material and methods

The culture of *T. carnegiei* CMW 52470 was obtained from the culture collection of the Forestry and Agricultural Biotechnology Institute (FABI) at the University of Pretoria and grown on malt extract agar (Merck, Wadeville, South Africa) at room temperature for approximately two weeks. DNA extraction proceeded as previously described for *Teratosphaeria* species (Wingfield et al. 2019). Sequencing took place at the Central Analytical Facilities (CAF), Stellenbosch University, using the Ion S5™ System and an Ion 530™ Chip (Thermo Fisher Scientific, MA, USA), at a target read length of 600 bp. After assessing read quality with FastQC 0.11.9 (Andrews 2010), the genome was assembled with SPAdes 3.15.2 (Bankevich et al. 2012), using the built-in read trimming function and kmer values of 21, 33, 55 77, 99 and 127. Genome completeness was assessed with BUSCO 4.1.4 (Simão et al. 2015), genome coverage was estimated by aligning the reads back to the genome with Bowtie 2.4.1 (Langmead and Salzberg 2012) and contamination was assessed with BlobToolKit 1.2 (Challis et al. 2020). Repeat content was determined with RepeatModeler 2.0.3 (Flynn et al. 2020) and open reading frames were predicted with the Funannotate 1.8.12 predict pipeline (Palmer and Stajich 2020).

The phylogenetic position of the sequenced strain relative to the other known *T. carnegiei* isolates and closely related *Teratosphaeria* species was determined using the ITS and beta-tubulin regions. The Maximum Likelihood tree was constructed by aligning the sequences with MAFFT v7.490 (Katoh and Standley 2013), manual alignment trimming and using ModelTest-NG 0.1.6 (Darriba et al. 2020) to identify the best nucleotide substitution model. Individual and concatenated gene trees were determined with RAxML-NG 1.1 (Kozlov et al. 2019), applying the transfer (TBE) bootstrap support of (Lemoine et al. 2018).

#### Results and discussion

Sequencing yielded 11.7 million reads ranging between 25 and 840 bp (mode = 532 bp) and FastQC did not flag any low-quality or overrepresented sequences. The final 27.69 Mb assembly had a coverage of approximately 150 X and comprised 1,135 contigs > 1 kb, with an L50 of 71 and an N50 of 128,647 bp. Genome completeness according to the Fungi_odb10 dataset was estimated at above 98% (745 complete BUSCOs = 98.3%) and BlobToolKit did not detect significant contamination. Funannotated predicted 9,464 protein-coding and 57 tRNA genes.

The 7.29% repetitive sequences identified in the *T. carnegiei* genome likely contributed to the low assembly contiguity. This proportion was less than half of the *ca.* 16–17% estimated for the assemblies of *T. destructans* CMW 44962 (Wingfield et al. 2018) and *T. eucalypti* CMW 54005 (Aylward et al. 2022), the two other *Teratosphaeria* species sequenced with the same technology. The *T. carnegiei* assembly, however, had better N50 and L50 values than either of those assemblies, further implying that the repeat content influenced the continuity of the assembly. The lower repeat content also influenced assembly size as the *T. carnegiei* genome was more than 2 Mb smaller than those of *T. destructans* CMW 44962 and *T. eucalypti* CMW 54005.

**Fig. 6:**
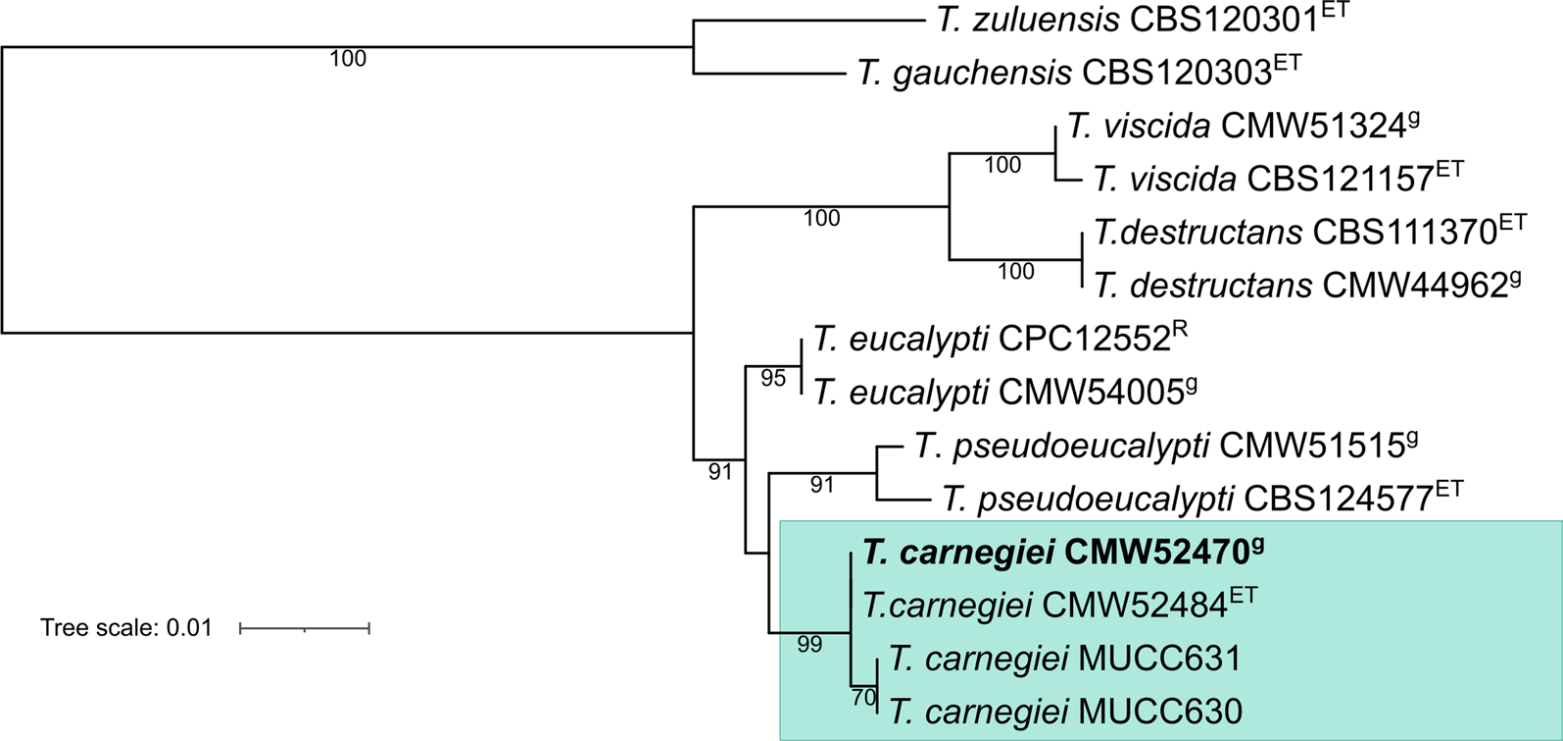
Maximum likelihood phylogeny of the concatenated ITS and beta-tubulin regions showing the phylogenetic position of *Teratosphaeria carnegiei* relative to other leaf pathogens in the tropical/subtropical clade. The stem pathogens *T. gauchensis* and *T. zuluensis* have been used as outgroups. Values on branches represent the transfer (TBE) bootstrap support. Superscripts indicate ex-type (ET), reference (R) and genome (g) strains. The strain sequenced in this study is shown in bold. GenBank accession numbers are available in Quaedvlieg et al. (2014), Aylward et al. (2019) and Crous et al. (2022).

Phylogenetic analyses of the ITS and beta-tubulin regions placed *T. carnegiei* within the lineage of tropical and subtropical leaf pathogens, where it shares a well-supported (91%) clade with *T. eucalypti* and *T. pseudoeucalypti* (Fig. 6). The relationship among these three cryptic species remains to be resolved, although the analysis of Andjic et al. (2010) suggests that *T. carnegiei* and *T. eucalypti* are sister species. All three taxa are known from diseased trees in eastern Australia plantations, but *T. eucalypti* and *T. pseudoeucalypti* are also known to cause disease problems beyond this range. For example, *T. eucalypti* is well -known in New Zealand (Hood et al. 2002) and *T. pseudoeucalypti* is important pathogen in South America (Cândido et al. 2014; Soria et al. 2014; Ramos and Pérez 2015). In contrast, the four *T. carnegiei* strains included in Fig. 6 are the only known isolates of this species, representing samples taken in 2009 (MUCC strains) and 2018 (CMW strains) from two plantations approximately 50 km apart (Andjic et al. 2010). A single point mutation in the beta-tubulin gene separates the isolates from these two sites.

The genome sequence of *T. carnegiei* brings the total number of sequenced *Teratosphaeria* species to nine. In addition to the aggressive tropical and subtropical foliar pathogens and the stem canker pathogens included in Figure 6, the species for which genomes have been sequenced include *T. nubilosa* which is an important pathogen of cold-tolerant *Eucalyptus* species such as *E. globulus* and *E. nitens* (Burgess and Wingfield 2017; Haridas et al. 2020), and the widely distributed but only mildly pathogenic *T. epicoccoides* (Taole et al. 2015; Havenga et al. 2020). Having genome sequences for these fungi will facilitate studies focused on better understanding their biology, disease management and global pathways of distribution.

*Authors*: Janneke Aylward*, Brenda D. Wingfield, Michael J. Wingfield.

*Contact: janneke@sun.ac.za.

## IMA GENOME‐F 18F

### Draft genome assembly of* Trichoderma atroviride* SC1, the biocontrol agent of grapevine pathogens

#### Introduction

*Trichoderma* is a genus of mainly asexual fungi belonging to the *Hypocreaceae*, primarily isolated from soils, roots, or leaves of plants present in every type of soil (tropical and temperate) (Howell 2002). These filamentous fungi present high genetic diversity and can be used to produce various products of commercial and ecological interest (Gupta et al. 2014). The benefits of *Trichoderma* species are well described in many sectors of industry and agriculture (Gupta et al. 2014).

*Trichoderma* species exert biocontrol against fungal phytopathogens using several mechanisms. *Trichoderma* can attack phytopathogens directly, using mechanisms such as mycoparasitism and antibiosis, or indirectly, competing for nutrients and space, or promoting plant growth and defense mechanisms (Sood et al. 2020; Vinale et al. 2008). *Trichoderma's* most salient characteristic is their ability to parasitize other fungi, which is ensured by a broad range of molecules, especially cell wall degrading enzymes (CWDEs) (Sood et al. 2020). Initially, *Trichoderma* uses transporters like the tripeptide transporter and the ABC transporter, to move towards a phytopathogenic fungus (Chet et al. 1981). Subsequently, *Trichoderma* produces innumerous CWDEs that hydrolyze the cellular walls of phytopathogenic fungi, ultimately leading to their death. Among CWDEs are chitinases, endochitinases, xylanases, proteases, and β-glucanases (Sharma et al. 2011). *Trichoderma* species, also present an antifungal arsenal that includes terpenes, pyrones, gliotoxin, gliovirin, and peptaibols, with activity against phytopathogens (Sharma et al. 2019; Vinale et al. 2008, 2020). When grouped together, antifungal molecules and CWDEs enhance their antibiotic effect against a broad spectrum of fungal phytopathogens (Tronsmo 1991). *Trichoderma* species can stimulate plant defenses, using molecules recognized as elicitors by the plant to trigger systemic defences (Hermosa et al. 2012; Lazazzara et al. 2021). Organic volatile compounds (VOCs), secondary metabolites in low concentrations, and phytohormone-like compounds produced by *Trichoderma* species can induce plant defenses, mainly salicylic acid, and ethylene-dependent defences (Hermosa et al. 2012; Lazazzara et al. 2021). *Trichoderma* can also increase plant root growth and productivity by influencing plant hormonal balance, increasing plant nutrient uptake, and solubilizing soil nutrients (Pozo et al. 2002; Sood et al. 2020). However, it is still unknown how these processes occur at a molecular level.

*Trichoderma atroviride* SC1 biocontrol potential against grapevine pathogens, responsible for several important diseases (i.e. grapevine trunk diseases or downy mildew), is well documented (Berbegal et al. 2020; Lazazzara et al. 2021; Leal et al. 2021; Martínez‐Diz et al. 2021; Pertot et al. 2017). In this study, we present the draft genome sequence of *Trichoderma atroviride* SC1 with the aim of advancing knowledge about this strain and its biocontrol potential against grapevine diseases. 

#### Sequenced strain

**Italy**: San Michele all'Adige, 46.1926600 N 11.1340928 E, isolated from *Corylus avellana* (CBS 122089) accession number through https://wi.knaw.nl/ and herbarium accession number through https://botzool.sci.muni.cz/herbarium:BRNU680030 (Savazzini et al. 2008).

#### Nucleotide sequence accession number

The draft genome of *Trichoderma atroviride* SC1 CBS strain 122089 reported here is made of high-quality assemblies. It has been deposited in GenBank under Acc. No. JAQOTD000000000 (BioProject No. PRJNA923860, assembly No. GCA_028554805.1, biosample No. SAMN32746547).

#### Materials and methods

The strain was cultivated from the commercial product Vintec® (Belchim crop Protection, Londerzeel, Belgium), was purified by single-spore isolation and maintained on potato dextrose agar (PDA) medium at 25 °C in the darkness. DNA was extracted with NucleoSpin Tissue (Macherey–Nagel, Duren, Germany), following the manufacturer’s protocol. Firstly, the complete ITS region, including the 5.8S gene, were amplified with ITS1/ITS4 (White et al. 1990), using the amplicon sequencing according to Eichmeier et al. (2010). The same DNA was used for genome library construction with the Nextera XT DNA Library Preparation Kit (Illumina, San Diego, USA). The library was sequenced using MiniSeq High Output Reagent Kit (300-cycles) (Illumina) with 2 × 150PE read option. The same DNA sample was sequenced using the Oxford Nanopore (LP-150), GridION FC (Oxford Nanopore Technologies, Oxford, UK), single-end, 1–200 kb reads, 5–10 Gb (DS-210). The sequence quality was checked using the FastQC-0.10.1 program (Andrews 2010). A FASTX-Toolkit Clipper (http://hannonlab.cshl.edu/fastx_toolkit/), specifying the Q33 parameter, was used to remove adaptors, and low-quality reads were discarded. Contigs of individual reads were assembled de novo using SPAdes genome assembler v. 3.15.2 (Prijibelski et al. 2020) with default settings, and a hybrid assembly of Illumina and nanopore reads was performed. The ab initio gene prediction was performed using Augustus (Keller et al. 2011) (-species = botrytis_cinerea -strand = both) for the assembled genome of *T. atroviride* SC1, resulting in predicted coding sequences. BUSCO 5.2.2 (Manni et al. 2021) revealed complete and single-copy proteins, posteriorly identified according to their function. Carbohydrate-active enzymes (CAZymes) were predicted using CAT and dbCAN3 servers (Yin et al. 2012). Signal peptides were detected by HMMER (Zhang and Wood 2003). Annotation was performed using JGI (Join Genome Institute). The search for secondary metabolite clusters was done using JGI MycoCosm. Placement of *T. atroviride* SC1 within the closest *Trichoderma* species (*Trichoderma Viride* clade) was verified using phylogenetic analysis of a ITS region. The dataset was aligned using the MAFFT v. 7 using the European Bioinformatics Institute platform (EMBL-EBI, https://www.ebi.ac.uk). Obtained alignment was manually checked and edited using Geneious Prime® 2023.1.1 (Biomatters, Inc., New Zealand). The maximum likelihood (ML) tree was constructed using IQ-TREE 2 (Minh et al. 2020). The best models for ML analyses were selected based on the Akaike Information Criterion (AIC) calculated in IQ-TREE 2. Trees were visualized in FigTree v. 1.4.4 and edited in Adobe Illustrator CC 2019.

#### Results and discussion

**Fig. 7:**
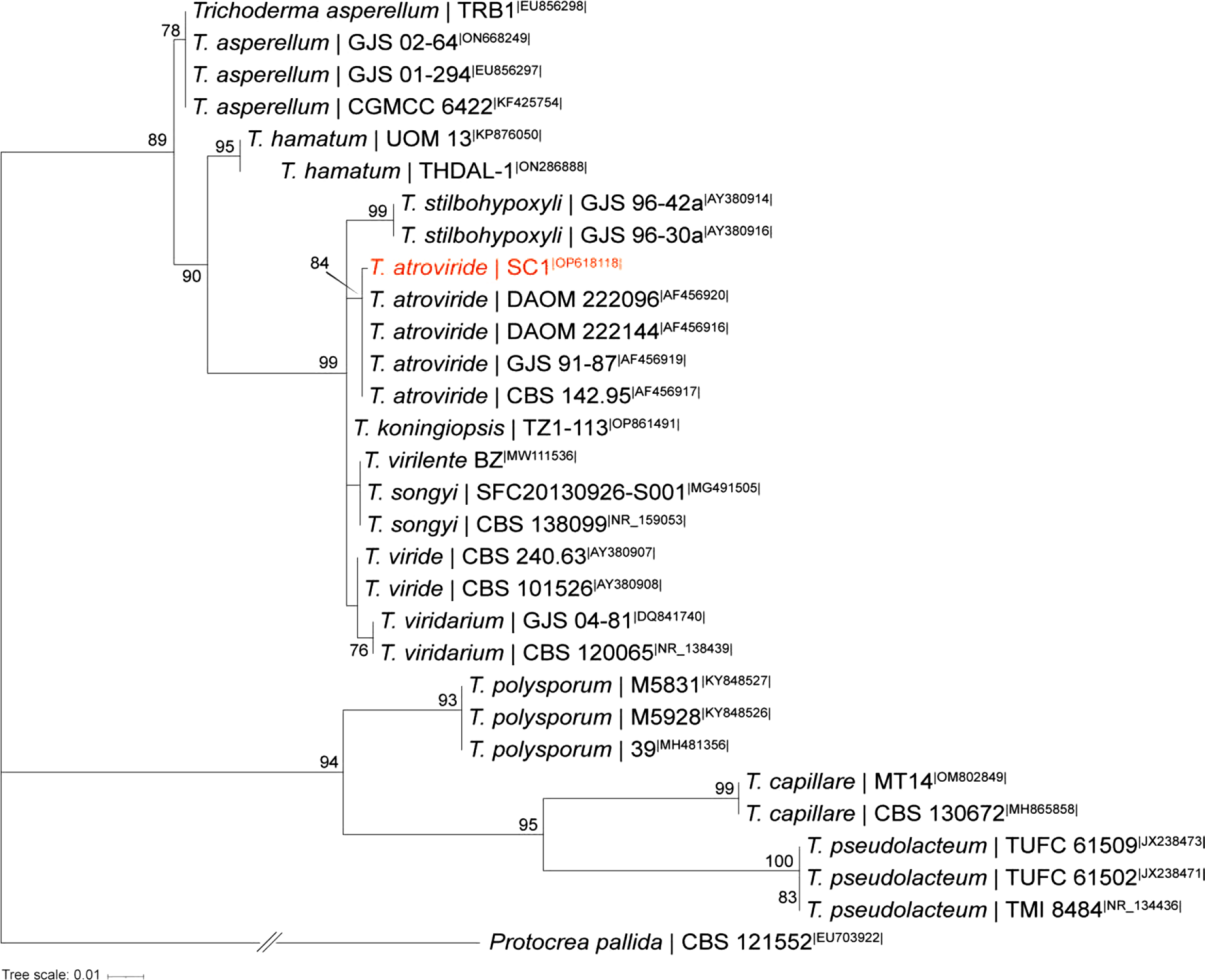
Maximum likelihood tree based on ITS region. Values at branch nodes are the bootstrapping confidence values with those ≥75% shown. The *Trichoderma atroviride* SC1 isolate sequenced in this study is indicted in red

Using Oxford Nanopore technology 1,503,165 reads were obtained with mean read length 4,918 bp. Sequencing by synthesis provided 14,630,016 reads and 13,771,719 reads passed the chastity filter. Genome coverage reached 50.5×. De novo assembly of *T. atroviride* SC1 CBS 122089 resulted in a genome size 35,757,960 bp with G + C content of 49.86%, and 603 contigs, with a scaffold length in which 50% of the total assembly length are covered (N50) values of 312,579 bp and the number contigs whose summed length is N50 (L50) of 35. The sequencing of ITS region (submitted to GenBank Acc. No. OP618118) confirmed a similarity score of 100% with *T. atroviride* available accessions, 545/545 nts. The phylogenetic placement of the genome is provided in Fig. 7. Genome completeness was estimated to be 97.2% corresponding to 96.8% complete and single-copy BUSCOs, 0.4% complete and duplicated BUSCOs and 2.2% missing BUSCOs. A total of 11,401 gene models were predicted in the *T. atroviride* SC1 assembly. Eighty-six signal peptides were detected by HMMER using dbCAN3. Signal peptides act as a zip codes, marking the protein secretion pathway as well as protein target location. In addition to protein targeting, a number of critical functions with or without regard to the passenger proteins have been attributed to signal peptides (Owji et al. 2018). A total of 129 CAZyme subfamilies were detected in 443 contigs using HMMER. The most represented CAZymes belonged to the subfamily (SBFs) GH18. Further classification of CAZymes based on their catalytic activity showed a high proportion of glycoside hydrolases (62 SBFs—48.1%), glycosyl transferases (30 SBFs—23.3%), carbohydrate-binding molecules (13 SBFs—10.1%), auxiliary activities (11 SBFs—8.5%), carbohydrate esterases (8 SBFs—6.2%), polysaccharide lyases (5 SBFs—3.9%). Compared to *T. afroharzianum* T11-W, *T. harzianum* CBS 266.95, *T. pleuroticola* (Zhou et al. 2020), or even *T. atroviride* IMI 206040 (Kubicek et al. 2011), *T. atroviride* SC1 has a high proportion of glycoside hydrolases. Using MicroStation Reader BioTek ELx808BLG (Biolog) and carbon sources (CS) in FF MicroPlate (Biolog Inc.), consumption was detected of 64 CS by *T. atroviride* SC1. This fungus was clearly identified as *T. atroviride* according to the FF MicroPlate database of Biolog Inc. Secondary metabolites are essential for fungal growth and development, providing protection against various stresses (Calvo et al. 2002). The search for secondary metabolite clusters revealed the presence of 38 clusters 12 × type I polyketide synthase, 11 × non-ribosomal peptide synthetase fragment, 8 × non-ribosomal peptide synthetase, 3 × terpene, 2 × polyketide-like and 2 × hybrid clusters.

In addition to *T. atroviride*, the genomic resource presented here includes seven other genomes (available from the National Center for Biotechnology Information) for this species associated with an effective biocontrol properties. The comparison of the available *T. atroviride* genome assemblies (Table 8) shows that the strain SC1 has the smallest genome and comparing to IMI 206040 and P1 strains has lower number of gene models. The availability of genomic resources for these fungi could facilitate and stimulate research aimed at resolving questions regarding their evolution, ecology, and, most importantly, their potential use in biocontrol.

**Table 8:** Genome statistics of the draft genome assemblies available for *Trichoderma atroviride* strains

Strain	GenBank Acc. No.	Size (Mb	No. of contigs	Gene models	ORFs/Mb	References
SC1	GCA_028554805.1	35.8	603	11401	318.5	Described here
JCM 9410	GCA_001599035.1	37.3	240	N.A.	N.A.	Horta et al. (2018)
IMI 206040	GCA_000171015.2	36.1	29	11810	327.1	Kubicek et al. (2011)
P1	GCA_020647795.1	37.3	7	13327	357.3	Li et al. (2021)
IMI 206040	GCA_019297715.1	36.2	12	N.A.	N.A.	N.A.
CG 6828	GCA_020466355.1	36.7	37	N.A.	N.A.	N.A.
LY357	GCA_002916895.1	35.9	637	N.A.	N.A.	N.A.
XS2015	GCA_000963795.1	36.4	357	N.A.	N.A.	Shi-Kunne et al. (2015)

*Table* 8 - *see additional TABLE*

*Authors*: Ales Eichmeier^*^, Eliska Hakalova, Jakub Pecenka, Milan Spetik, Catarina Leal, David Gramaje^*^

*Contact: ales.eichmeier@mendelu.cz; david.gramaje@icvv.es.

### Supplementary information

**Additional file 1. Table 1.** Summary of genomes analysed during this study that were correctly identified.

## Data Availability

Genome data for the *Penicillium* genomes are publicly available in the NCBI genome database (https://www.ncbi.nlm.nih.gov/datasets/genome). The datasets generated from *Trichoderma atroviride* during the current study are available in the NCBI repository, https://www.ncbi.nlm.nih.gov/bioproject/923860. For the *Penicillium* species from dry cured meat the genome assembly and annotations are available from JGI Fungal genome portal MycoCosm under JGI Projects: 1,289,827 (ITEM 15300), 1,289,819 (ITEM 18316), 1,289,903 (ITEM 18327), and have been deposited to GenBank under BioProjects: PRJNA970850 (ITEM 15300), PRJNA971651 (ITEM 18316), PRJNA970851 (ITEM 18327). Genome assembly and annotations are available from JGI Fungal genome portal MycoCosm under JGI Project Id 1,289,847 and has been deposited to GenBank under BioProject n.PRJNA971650 (BioSample n. SAMN35051277; Project Accession n. SRP442271). The genomic sequences of *T. carnegiei* have been deposited at DDJ/EMBL/GenBank under the accession JANYMD000000000. This paper describes the first version. The genomes of the *Pewenomyces* species have been deposited in the NCBI genome database.
